# Factors influencing necrotizing enterocolitis in premature infants in China: a systematic review and meta-analysis

**DOI:** 10.1186/s12887-024-04607-3

**Published:** 2024-02-29

**Authors:** Shuliang Zhao, Huimin Jiang, Yiqun Miao, Wenwen Liu, Yanan Li, Hui Liu, Aihua Wang, Xinghui Cui, Yuanyuan Zhang

**Affiliations:** 1 School of Nursing, Shandong Second Medical University, Weifang, 261053 China; 2 Nursing Department Affiliated Hospital of Shandong Second Medical University, Weifang, 261031 China; 3 School of Nursing, Capital Medical University, Beijing, 100071 China; 4 Xiangya School of Nursing, Central South University, Changsha, 410000 China

**Keywords:** Necrotizing enterocolitis, Premature infant, Influencing factors, Risk factors, Meta-analysis, China

## Abstract

**Background:**

Necrotizing enterocolitis (NEC) is a multifactorial gastrointestinal disease with high morbidity and mortality among premature infants. However, studies with large samples on the factors of NEC in China have not been reported. This meta-analysis aims to systematically review the literature to explore the influencing factors of necrotizing enterocolitis in premature infants in China and provide a reference for the prevention of NEC.

**Methods:**

PubMed, Embase, Web of Science, Cochrane Library, China National Knowledge Infrastructure (CNKI), China Biomedical Literature Database (CBM), Wanfang and VIP databases were systematically searched from inception to February 2023. We used Stata14.0 software to perform the systematic review and meta-analysis. We used fixed or random effects models with combined odds ratios (ORs) and 95% confidence intervals (CIs), and quality was evaluated using the Newcastle‒Ottawa Scale (NOS).

**Results:**

The total sample was 8616 cases, including 2456 cases in the intervention group and 6160 cases in the control group. It was found that 16 risk factors and 3 protective factors were related to necrotizing enterocolitis in premature infants. Septicemia (OR = 3.91), blood transfusion (OR = 2.41), neonatal asphyxia (OR = 2.46), pneumonia (OR = 6.17), infection (OR = 5.99), congenital heart disease (OR = 4.80), intrahepatic cholestasis of pregnancy (ICP) (OR = 2.71*)*, mechanical ventilation (OR = 1.44), gestational diabetes mellitus (GDM) (OR = 3.08), respiratory distress syndrome (RDS) (OR = 3.28), hypoalbuminemia (OR = 2.80), patent ductus arteriosus (PDA) (OR = 3.10), respiratory failure (OR = 7.51), severe anemia (OR = 2.86), history of antibiotic use (OR = 2.12), and meconium-stained amniotic fluid (MSAF) (OR = 3.14) were risk factors for NEC in preterm infants in China. Breastfeeding (OR = 0.31), oral probiotics (OR = 0.36), and prenatal use of glucocorticoids (OR = 0.38) were protective factors for NEC in preterm infants.

**Conclusions:**

Septicemia, blood transfusion, neonatal asphyxia, pneumonia, infection, congenital heart disease, ICP, GDM, RDS, hypoproteinemia, PDA, respiratory failure, severe anemia, history of antibiotic use and MSAF will increase the risk of NEC in premature infants, whereas breastfeeding, oral probiotics and prenatal use of glucocorticoids reduce the risk. Due to the quantity and quality of the included literature, the above findings need to be further validated by more high-quality studies.

**Supplementary Information:**

The online version contains supplementary material available at 10.1186/s12887-024-04607-3.

## Introduction

Necrotizing enterocolitis (NEC) is one of the most common gastrointestinal diseases in neonates [1]. The onset of NEC is not clinically specific, and the disease progresses rapidly and can be complicated by intestinal perforation and necrosis, causing death in severe cases [2]. Surviving children may experience severe sequelae, including intestinal stricture, short bowel syndrome, dependence on total parenteral nutrition, and neurodevelopmental disorders, leading to both growth and mental retardation. These conditions significantly impact the quality of life in later years [3, 4]. Over the past few years, China has made great progress in perinatal and neonatal intensive care, the survival rate has increased, and the number of premature infants has increased sharply, especially very low birth weight (VLBW) or extremely premature infants at high risk of NEC, which reflects the importance of studying the epidemiology of contemporary NEC [5, 6]. In China, the incidence of NEC was found to be 5.5% and 7.0% for infants with birth weights < 1500 g and < 1000 g, respectively, and 4.8% and 7.6% for infants born at < 32 weeks and < 28 weeks, respectively [7]. Therefore, NEC remains a fulminant disease, necessitating improved prevention, early diagnosis, and more rational management.

NEC results from a combination of factors with complex pathophysiology and unclear mechanisms, and individual risk factors remain to be elucidated [8]. Identifying the clinical features that can predict NEC from the many influencing factors is the focus of prevention. Both maternal and neonatal factors may be risk factors for the development of NEC. It has been found that early identification of NEC risk factors, targeted interventions, and timely diagnosis and treatment are extremely important to reduce the morbidity and mortality of NEC [9]. Currently, relevant studies on the influencing factors of NEC in Chinese preterm infants have been reported [10–12]. However, the influence of the sample size and the completeness of the children’s data has led to considerable variability in the results of these studies. As a result, the specific risk factors and protective factors related to NEC remain unclear. Although overseas studies on factors affecting NEC in preterm infants have been conducted for a long time [13, 14], differences in medical practices, geographic environments, and demographics between regions may limit the blind generalization of known findings to all areas.

Although meta-analyses of risk factors for neonatal NEC have been reported [15–17], opinions differ on the importance of NEC risk factors. The opinion of a panel of 35 international NEC experts pointed to the ambiguity of risk factors affecting NEC except for gestational age (GA), birth weight (BW) and feeding [18]. Studies are limited by differences in population characteristics and different definitions of NEC, leading to some variability in findings. This suggests that there are modifiable risk factors that give healthcare professionals the opportunity to intervene to reduce the risk of NEC. Considering that there is no meta-analysis for the factors affecting NEC in Chinese preterm infants, this study used systematic evaluation and meta-analysis by searching the latest literature to explore the major risk factors and protective factors affecting the occurrence of NEC in preterm infants in China, and to provide a scientific basis for the development of preventive measures for NEC.

## Materials and methods

### Search strategy

The present systematic review and meta-analysis followed the preferred reporting items in the systematic review and meta-analysis (PRISMA) guidelines [19]. The PRISMA checklist is presented in S Table 1. The databases of China National Knowledge Infrastructure (CNKI), VIP (Chinese) and WanFang (Chinese) database, China Biomedical Literature Database (CBM), Web of Science, Embase, and PubMed were searched for case‒control studies and cohort studies from creation to February 2023. We searched the databases using a combination of subject terms and free words, while references of included studies were hand-searched to supplement the relevant information obtained. The keywords included (“premature infant” OR “preterm infants “OR” Neonatal Prematurity” OR “very preterm infant” OR” extremely preterm infants” OR “Very low birth weight”) AND (“necrotizing enterocolitis”) AND (“risk factor” OR “influence factors”) AND (“ case‒control studies” OR “cohort study”) AND (“Chinese” OR “China”). Detailed information on the search terms and search strategies is shown in S Table 2.

### Inclusion and exclusion criteria

*Inclusion criteria*: (1) Study participants: Chinese preterm infants with NEC, GA < 37 weeks, weight < 2500 g; (2) Clear diagnosis of NEC, defined as stage II and above according to the Bell criteria, with gastrointestinal dysfunction clearly demonstrated by clinical symptoms and imaging assessment [20] (3). Study type: case-control or cohort study. The intervention group in the case-control study was preterm infants with confirmed NEC, and the control group was preterm infants without NEC; the exposure group in the cohort study was preterm infants exposed to risk factors associated with NEC, and the control group was preterm infants not exposed to these risk factors (4). Study outcome: Risk factors and protective factors affecting NEC in preterm infants (5). Study language: Published in English or Chinese (6). Study date: Each database from the establishment to February 2023.

*Exclusion criteria*: (1) conference abstracts, case reports, review categories, and literature without control groups; (2) literature with incomplete original study data and an inability to extract data; (3) Studies with duplicate publication.

### Data extraction and quality assessment

Three researchers independently extracted data according to the developed data collection form, and in case of discrepancies, the three discussed and approved. Data extracted included author, publication year, study area, study time, study type, study population, NEC diagnostic criteria, number of cases/control groups, influencing factors and Newcastle‒Ottawa Scale (NOS) score. Two researchers independently performed quality assessment using the NOS recommended by the Cochrane Collaboration Network [21]. In the event of disagreements, group discussions were held to reach a consensus. This scale includes 3 modules of study population, comparability between groups, and outcome evaluation and is divided into 8 entries with a score out of 9. NOS score ≥ 6 is considered high-quality literature. We assessed the methodological quality of our systematic reviews using the A Measurement Tool to Assess Systematic Reviews 2 (AMSTAR2) [22]. The details of the items in the AMSTAR-2 tool are shown in S Table 3.

### Data synthesis and statistical analysis

We used Stata 14.0 software to perform statistical analysis of the extracted data. Heterogeneity among the studies was analyzed using the χ^2^ test (test level α = 0.1), and the Q test was combined with the *I*^*2*^ statistic to determine heterogeneity. When *I*^*2*^ ≥ 50%, it indicated high heterogeneity among the study results, and a random-effects model was used. Otherwise, the fixed effects model was used. To assess the stability of the combined results for statistically significant risk factors, we conducted sensitivity analysis by comparing the values obtained from both the fixed-effects model and the random-effects model. Funnel plots were used to assess publication bias, and Egger’s statistical test was used to analyze whether publication bias was statistically significant. Differences were considered statistically significant at *P* < 0.05.

## Results

### Study selection

**Fig. 1:**
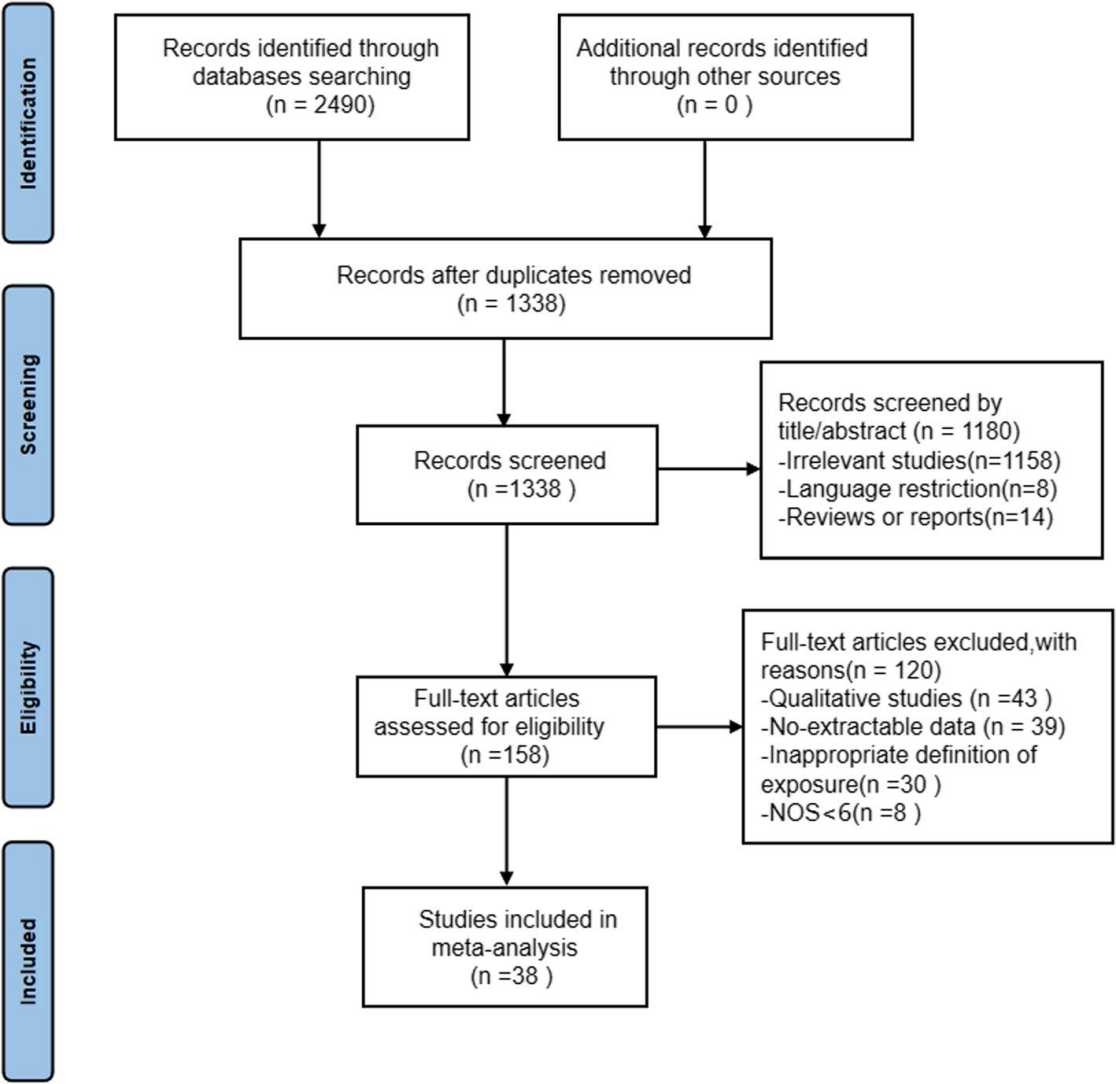
Flow chart depicting the studies screened, selected and included based on PRISMA. NOS: Newcastle‒Ottawa Scale. PRISMA: Preferred Reporting Items for Systematic Reviews and Meta-Analyses

The initial search yielded 2490 articles, and 1152 duplicates were removed after screening by Endnote software; 1180 articles were removed after reading the title and abstract; 158 articles were retained for the full text screening, and 38 eligible articles [10–12, 23–57] were finally included. No additional eligible studies were identified through a manual search. The study selection flow chart is shown in Fig. 1.

### Study characteristics and qualit y

Thirty-eight studies (36 case‒control studies [10–12, 23–54, 57] and 2 cohort studies [55, 56]) were included, containing a total sample size of 8616 cases, with 2456 cases in the intervention group and 6160 cases in the control group, from 16 different provinces in China. The study population consisted of preterm infants with BW less than 2500 g, GA less than 37 weeks, and Bell staging ≥ II. Diagnostic criteria for NEC vary. Twenty-one studies utilized the revised Bell Subdivision periodical standard of Practical Neonatology, 4th edition, for the diagnosis of NEC in preterm infants [58]. Two studies referred to the revised Bell Subdivision periodical standard of Practical Neonatology, 5th edition [59]. Eight studies used the modified BELL-NEC grading scale [20]. Two studies used the revised Bell Subdivision periodical standard [60]. Three studies referred to Avery’s Diseases of the Newborn as the revised Bell marking criteria [61]. One study adopted to the Vermont Oxford Network’s revised diagnostic criteria based on Bell staging [62]. One study referred to the U.S. Guidelines for the Management of Necrotizing Small Bowel Colitis in Very Low Birth Mass Children [63]. The detailed characteristics are shown in Table 1. Nine studies received a NOS quality assessment score of 6, 15 studies scored 7, 9 studies scored 8, and 5 studies scored 9. The overall quality of the studies was above the average (S Table 4).

**Table 1:** Basic characteristics of research and the evaluation of research quality

Author, year	Study area	Study time	Study type	Study population	NEC diagnostic criteria	Case group/ control group	Influencing factors	NOS score
Zeng, 2021 [24]	Sichuan	2011.1-2019.10	Case control	BW < 1500 g, Bell staging ≥ II	Adoption of modified BELL-NEC grading standards (1978)	30/467	①⑮	7
Cheng, 2016 [23]	Sichuan	2010.3-2015.3	Case control	GA < 34 weeks, Bell staging ≥ II	Practical Neonatology, 4th Revised Bell staging diagnostic criteria	66/132	①②③⑨⑫	8
Liu, 2019 [25]	Fujian	2012.1-2018.12	Case control	BW < 2500 g	Practical Neonatology, 4th Revised Bell staging diagnostic criteria	95/95	④⑦⑯	7
Shang, 2014 [26]	Sichuan	2011.8-2013.9	Case control	GA < 37 weeks, BW < 2500 g	Adoption of modified BELL-NEC grading standards (1978)	37/62	④⑨⑩	7
Sun, 2017 [27]	Shandong	2013.12-2016.9	Case control	GA < 37 weeks, BW < 2000 g	Practical Neonatology, 4th Revised Bell staging diagnostic criteria	37/60	②③⑤⑨⑩	8
Dong, 2021 [10]	Henan	2012.1-2019.11	Case control	GA < 32 weeks, Bell Staging II-III	Revised Bell Subdivision periodical standard (1986)	113/113	①⑩	7
Lu, 2022 [28]	Zhejiang	2018.1-2019.12	Case control	GA < 34weeks, BW < 1500 g, Bell staging ≥ II	Practical Neonatology, 5th Edition	52/52	②⑨⑬	6
Wang, 2022 [29]	Jilin	2011.1-2020.12	Case control	GA < 37weeks, Bell Staging II-III	Practical Neonatology, 5th Edition	298/300	②⑨	7
Li, 2019 [30]	Shanxi	2012.2-2018.11	Case control	GA < 37 weeks, BW < 2500 g	Practical Neonatology, 4th Revised Bell staging diagnostic criteria	25/175	③⑤⑥⑨⑩⑰	6
Lu, 2018 [31]	Guangxi	2013.1-2015.5	Case control	GA < 33weeks, Bell staging ≥ II	Practical Neonatology, 4th Revised Bell staging diagnostic criteria	50/50	①③⑨⑩⑪⑱	7
Lu, 2013 [32]	Jiangxi	2011.7-2013.4	Case control	GA < 37 weeks, BW < 2500 g	Refer to Avery’s diseases of the newborn the revised Bell marking criteria	54/57	①④⑰	6
Lu, 2015 [33]	Xamen	2008.1-2011.12	Case control	GA < 37 weeks, BW < 2500 g	Practical Neonatology, 4th Revised Bell staging diagnostic criteria	59/95	④⑨⑩	6
Qu, 2019 [34]	Guangdong	2013.6-2015.12	Case control	GA ≤ 32weeks	Practical Neonatology, 4th Revised Bell staging diagnostic criteria	65/130	①②③⑦⑧⑫	8
Shi, 2019 [35]	Shanxi	2015.1-2018.6	Case control	GA < 37 weeks, BW < 2500 g	Practical Neonatology, 4th Revised Bell staging diagnostic criteria	32/150	①②③⑦⑧⑩	7
Wang, 2014 [36]	Beijing	2010.10-2012.12	Case control	GA < 33weeks, Bell staging ≥ II	Adoption of modified BELL-NEC grading standards (1978)	49/121	③	7
Yu, 2018 [37]	Guizhou	2006.1-2015.12	Case control	BW < 2500 g	Practical Neonatology, 4th Revised Bell staging diagnostic criteria	146/146	①②⑨⑩⑪	7
Zhu, 2012 [38]	Jiangsu	2006.8-2011.4	Case control	BW < 1500 g, Bell staging ≥ II	Practical Neonatology, 4th Revised Bell staging diagnostic criteria	20/303	①⑪	8
Hou, 2017 [39]	Liaoning	2011.1-2016.1	Case control	GA < 37 weeks, BW < 2500 g	Practical Neonatology, 4th Revised Bell staging diagnostic criteria	76/80	①⑧⑨	7
Song, 2021 [40]	Henan	2012.1-2020.7	Case control	BW < 2500 g, Bell Staging II-III	Revised Bell Subdivision periodical standard (1986)	166/166	②⑱	9
Zhang, 2019 [41]	Sichuan	2013.1-2016.12	Case control	GA< 37weeks, BW 1 000 ~ 1 499 g	Practical Neonatology, 4th Revised Bell staging diagnostic criteria	33/33	①③⑩	8
Lu, 2017 [42]	Chongqing	2010.3-2015.3	Case control	GA < 37 weeks, BW < 2500 g, Bell staging ≥ II	Practical Neonatology, 4th Revised Bell staging /diagnostic criteria	66/132	①②③⑨⑫	9
Zhu, 2021 [43]	Anhui	2015.5-2020.1	Case control	BW ≥ 1000 g, < 1500 g, Bell staging ≥ II	Refer to Avery’s diseases of the newborn the revised Bell marking criteria	19/218	①⑨⑳	9
Huang, 2022 [44]	Chongqing	2008.1-2021.12	Case control	small for GA infant, prematurity, Bell staging ≥ II	Reference to the Vermont Oxford Network’s revised diagnostic criteria based on Bell staging	140/280	①⑨⑬⑱	7
Liu, 2022 [11]	Neimenggu	2015.1-2021.12	Case control	GA < 32weeks	Reference Modification Bell Staging (1978)	77/577	①③⑮⑱	8
Yang, 2022 [45]	Beijing	2016.3-2020.6	Case control	GA < 37 weeks, BW < 2500 g,	Reference Modification Bell Staging (1978)	78/100	①⑬	8
Yang, 2018 [46]	Sichuan	2016.1-2016.12	Case control	GA < 37 weeks	Practical Neonatology, 4th, Revised Bell staging diagnostic criteria	22/407	⑧⑩⑬⑭	7
Zhuang, 2007 [47]	Fujian	2002.1-2005.5	Case control	GA < 37 weeks	Refer to Avery’s diseases of the newborn the revised Bell marking criteria	20/80	①⑨⑭	6
Li ,2020 [48]	Jiangsu	2014.5-2018.12	Case control	GA 32–37 weeks, BW < 2500 g	Reference to the U.S. Guidelines for the Management of Necrotizing Small Bowel Colitis in Very Low Birth Mass Children	57/30	②③⑯	7
Ma, 2021 [49]	Zhejiang	2017.9-2020.6	Case control	GA < 34weeks, Bell staging ≥ stage II	Reference Modification Bell Staging (1978)	54/106	⑥⑫	6
Deng, 2017 [50]	Sichuan	2014.1-2016.12	Case control	GA < 34weeks, Bell staging ≥ stage II	Practical Neonatology, 4th Revised Bell staging diagnostic criteria	54/108	①②③⑦⑬	6
Zhu, 2020 [51]	Hebei	2019.1-2019.9	Case control	GA < 37 weeks, BW < 2500 g	Practical Neonatology, 4th Revised Bell staging diagnostic criteria	38/462	①③④⑨⑩	8
Wang, 2017 [52]	Henan	2013.6-2016.6	Case control	GA < 37 weeks, BW < 2500 g	Practical Neonatology, 4th Revised Bell staging diagnostic criteria	58/36	①⑦⑨⑩	7
Wang, 2020 [53]	Henan	2010.1-2018.12	Case control	GA < 37 weeks, BW < 2500 g	Practical Neonatology, 4th Revised Bell staging diagnostic criteria	30/34	①④⑨⑩	6
Zhang, 2017 [54]	Guangdong	2013.1-2015.12	Case control	GA < 32 weeks	Reference Modification Bell Staging (1978)	61/376	①③⑩⑳	6
Chen, 2020 [55]	Chongqing	2010.1-2016.10	Cohort study	GA < 34 weeks, Bell staging ≥ stage II	Practical Neonatology, 4th Revised Bell staging diagnostic criteria	30/150	①⑲	8
Tan, 2022 [56]	Sichuan	2019.1-2021.6	Cohort study	1500 g < BW < 2500 g	Practical Neonatology, 4th Revised Bell staging diagnostic criteria	68/124	①②⑩⑪	9
Yu, 2023 [12]	Hainan	2014.1-2021.12	Case control	BW < 1500 g, Bell staging ≥ stage II	Reference Modification Bell Staging (1978)	62/62	①⑯⑱	9
Tain, 2023 [57]	Shandong	2017.4-2019.6	Case control	GA < 37 weeks, BW < 1500 g	Practical Neonatology, 4th Revised Bell staging diagnostic criteria	19/91	①③⑨⑲	7

*GA* gestational age, *BW* birth weight, *NEC* necrotizing enterocolitis, *NOS* Newcastle-Ottawa Scale

①Septicemia ②Blood transfusion ③Neonatal asphyxia ④Pneumonia ⑤Infection occurs ⑥Gestational diabetes ⑦Respiratory distress syndrome ⑧Mechanical ventilation ⑨Oral probiotics ⑩Breastfeeding ⑪Congenital heart disease ⑫Intrahepatic cholestasis of pregnancy ⑬Prenatal application of glucocorticoids ⑭Intravenous immunoglobulin ⑮Hypoalbuminemia ⑯Patent ductus arteriosus ⑰respiratory failure ⑱Severe anemia ⑲Meconium-stained amniotic fluid ⑳History of antibiotic use

### Meta-analysis results

The heterogeneity analysis for factors influencing NEC in preterm infants revealed that septicemia, blood transfusion, neonatal asphyxia, pneumonia, infection occurrence, intrahepatic cholestasis of pregnancy (ICP), mechanical ventilation, gestational diabetes mellitus (GDM), respiratory distress syndrome (RDS), prenatal application of glucocorticoids, hypoproteinemia, ductus arteriosus, severe anemia, and meconium-stained amniotic fluid (MSAF) exhibited low heterogeneity (*I*^*2*^ < 50%) when analyzed using a fixed-effects model with combined effect sizes. On the other hand, oral probiotics, breastfeeding, congenital heart disease, respiratory failure, and history of antibiotic use had high heterogeneity (*I*^*2*^ ≥ 50%) and were analyzed using a random-effects model. The meta-analysis indicated that intravenous immunoglobulin was not statistically significant with NEC in premature infants (*P* > 0.05), while all other influencing factors were statistically significant. We divided the factors that affect preterm infants with NEC into two main categories: protective factors and risk factors. Further details are provided in Table 2.

**Table 2:** Heterogeneity test and meta-analysis results of NEC risk factors in premature infants

Research factors	Number of studies	Heterogeneity test			Effect model	Pooled OR (95%CI)	Pooled *p* value
		Q	I^2^ (%)	*P*			
Septicemia	25	43.43	45	0.009	Fixed effect	3.91 (3.37, 4.55)	< 0.001
Blood transfusion	13	23.68	49	0.02	Fixed effect	2.41 (1.97, 2.95)	< 0.001
Neonatal asphyxia	14	23.58	45	0.04	Fixed effect	2.46 (2.07, 2.93)	< 0.001
Pneumonia	6	1.34	0	0.93	Fixed effect	6.17 (3.98, 9.57)	< 0.001
Infection occurs	2	1.43	30	0.23	Fixed effect	5.99 (2.57, 13.93)	< 0.001
Oral probiotics	17	96.90	83	< 0.001	Random effect	0.36 (0.25, 0.53)	< 0.001
Breastfeeding	13	302.01	96	< 0.001	Random effect	0.31 (0.16, 0.62)	< 0.001
Congenital heart disease	3	2.01	1	0.37	Fixed effect	4.80 (3.00, 7.68)	< 0.001
Intrahepatic cholestasis of pregnancy	4	2.17	0	0.54	Fixed effect	2.71 (1.92, 3.82)	< 0.001
Mechanical ventilation	4	0.68	0	0.88	Fixed effect	1.44 (1.22, 1.71)	< 0.001
Gestational diabetes	2	0.06	0	0.80	Fixed effect	3.08 (1.73, 5.48)	0.0001
Respiratory distress syndrome	4	1.24	0	0.74	Fixed effect	3.28 (2.23, 4.85)	< 0.001
Prenatal application of glucocorticoids	5	6.19	35	0.19	Fixed effect	0.38 (0.24, 0.60)	< 0.001
Intravenous Immunoglobulin	2	5.46	82	0.02	Random effect	0.70 (0.10, 5.20)	0.73
Hypoalbuminemia	2	0.43	0	0.51	Fixed effect	2.80 (1.78, 4.41)	< 0.001
Patent ductus arteriosus	3	0.28	0	0.87	Fixed effect	3.10 (1.93, 4.98)	< 0.001
respiratory failure	2	3.50	71	0.06	Random effect	7.51 (1.60, 35.10)	0.01
Severe anemia	4	2.02	0	0.57	Fixed effect	2.86 (2.06, 3.99)	< 0.001
History of antibiotic use	2	4.53	78	0.03	Random effect	2.12 (1.18, 3.81)	0.01
Meconium-stained amniotic fluid	2	0.05	0	0.82	Fixed effect	3.14 (1.64, 6.01)	< 0.001

### Protective factors

This study showed that breastfeeding [OR = 0.31, 95% CI (0.16, 0.62), *P* < 0.001], oral probiotics [OR = 0.36, 95% CI (0.25, 0.53), *P* < 0.001] and prenatal application of glucocorticoids [OR = 0.38, 95% CI (0.24, 0.60), *P* < 0.001] were protective factors for NEC in preterm infants.

### Risk factors

Septicemia [OR = 3.91, 95% CI (3.37,4.55), *P* < 0.001], blood transfusion [OR = 2.41, 95% CI (1.97, 2.95), *P* < 0.001], severe anemia [OR = 2.86, 95% CI (2.06, 3.99), *P* < 0.001], neonatal asphyxia [OR = 2.46, 95% CI (2.07, 2.93), *P* < 0.001], pneumonia [OR = 6.17, 95% CI (3.98, 9.57), *P* < 0.001], mechanical ventilation [OR = 1.44, 95% CI (1.22, 1.71), *P* < 0.001], RDS [OR = 3.28, 95% CI (2.23,4.85), *P* < 0.001], congenital heart disease [OR = 4.80 95% CI (3.00, 7.68), *P* < 0.001], hypoproteinemia [OR = 2.80, 95% CI (1.78, 4.41), *P* < 0.001], PDA [OR = 3.10, 95% CI (1.93, 4.98), *P* < 0.001], history of antibiotic use [OR = 2.12, 95% CI (1.18,3.81), *P* = 0.01], infection occurs [OR = 5.99, 95% CI (2.57, 13.93), *P* < 0.001], ICP [OR = 2.71, 95% CI (1.92, 3.82), *P* < 0.001], GDM [OR = 3.08, 95% CI (1.73, 5.48), *P* < 0.001], respiratory failure [OR = 7.51, 95% CI(1.60, 35.10), *P* = 0.01], MSAF [OR = 3.14, 95% CI (1.64, 6.01), *P* < 0.001] were risk factors for NEC in preterm infants.

 Due to the multitude of influential factors considered, forest plots for septicemia and oral probiotics are presented in this paper (Figs. 2 and 3). Forest plots of other influencing factors are provided in S Figs. 1–18.

**Fig. 2:**
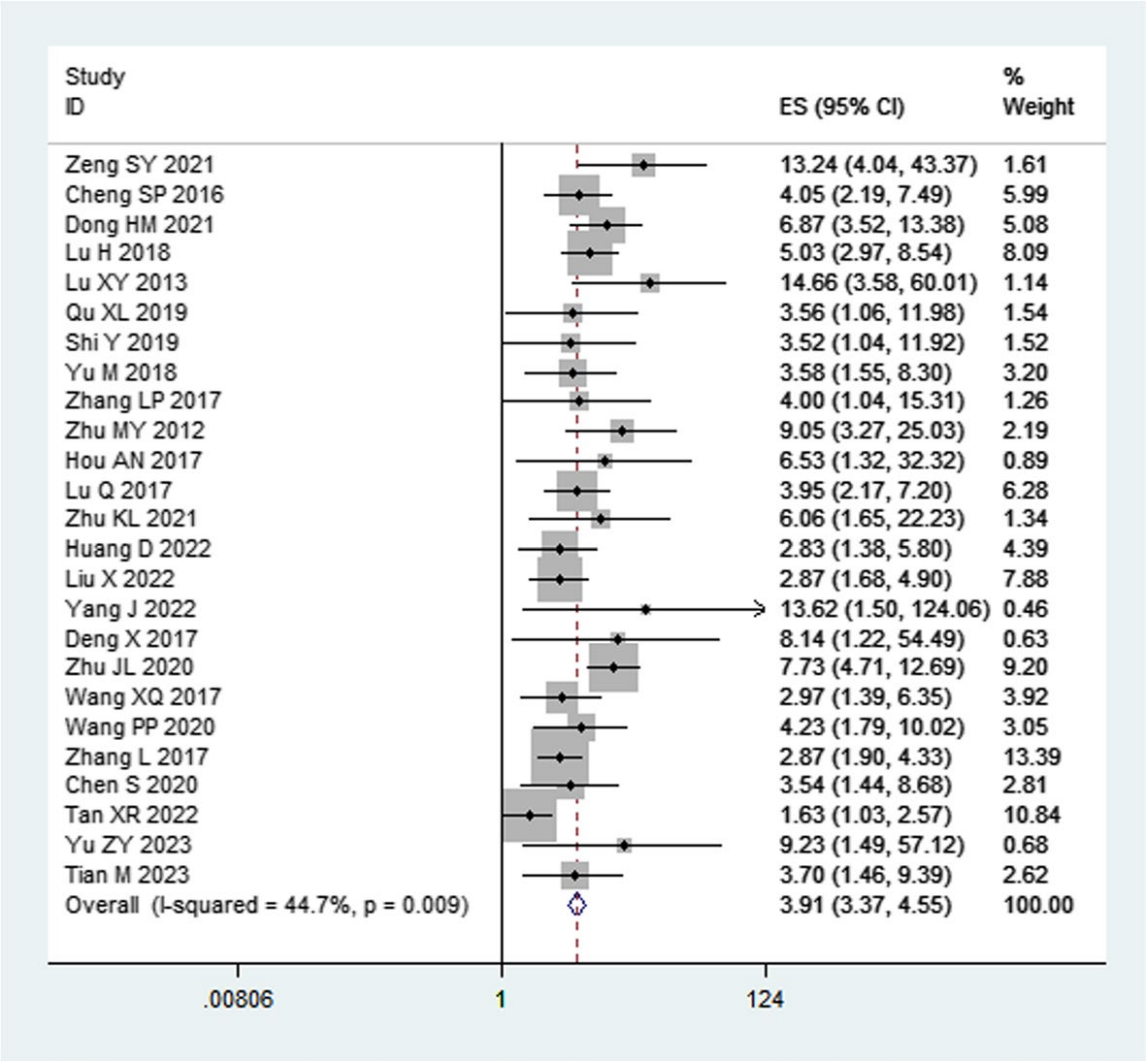
Forest plot of septicemia as a risk factor analysis for NEC preterm infants. NEC: necrotizing enterocolitis; ES: Odds ratio (OR)

**Fig. 3:**
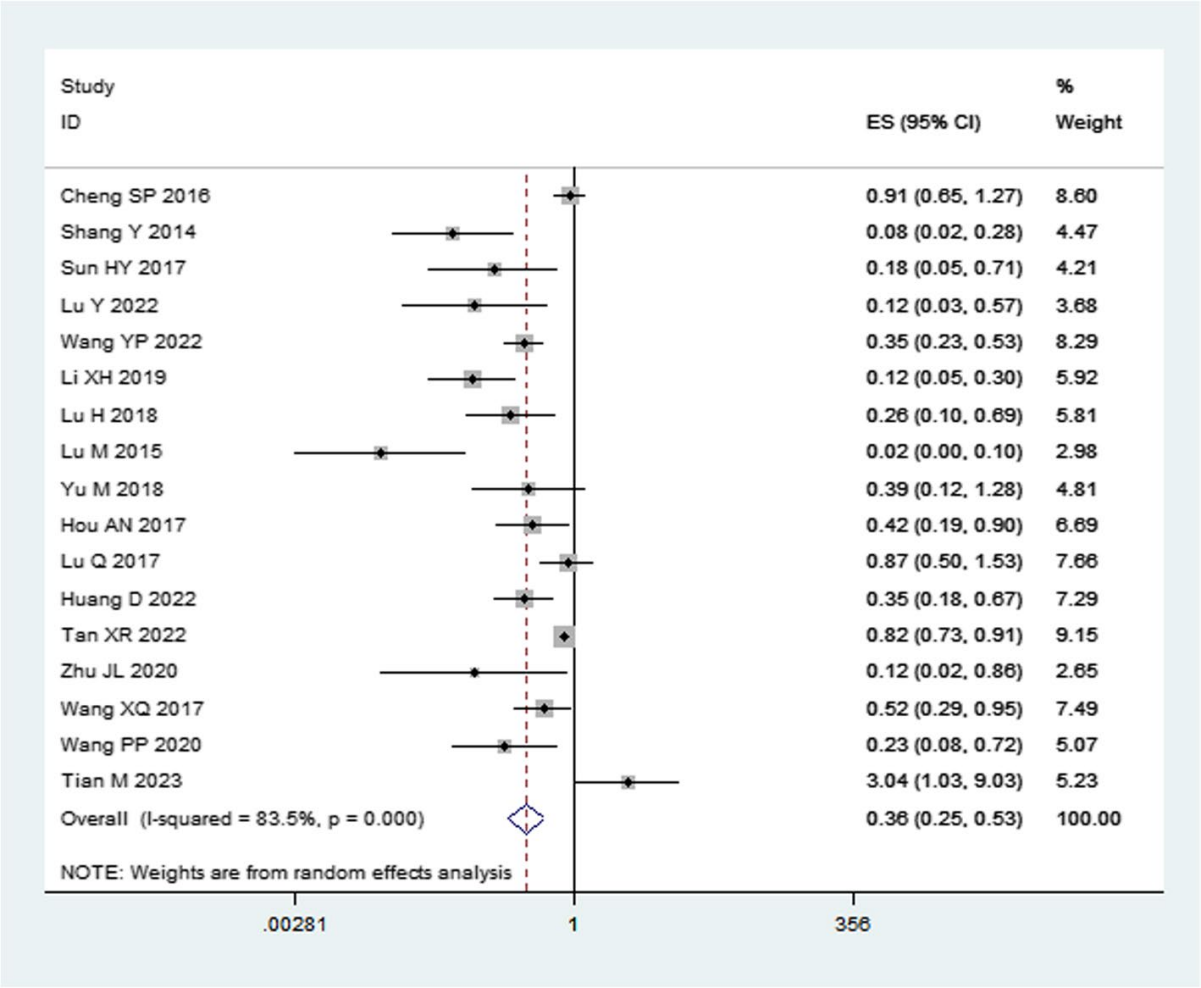
Forest plot for analysis of oral probiotics as a protective factor for NEC preterm infants. NEC: necrotizing enterocolitis; ES: Odds ratio (OR)

### Sensitivity analysis and publication bias

Sensitivity analyses were performed by eliminating each study individually, and the remaining articles were recombined for meta-analysis. Sensitivity analyses revealed that the heterogeneity for studies with high heterogeneity, including oral probiotics, breastfeeding, respiratory failure, and history of antibiotic use, remained consistent after removing any individual study, suggesting that the results of the meta-analysis were relatively stable. The study by Tan et al. [56] had a large impact on the outcome of preterm heart disease and was the main source of heterogeneity. Upon excluding this study and reanalyzing the results, a substantial reduction in heterogeneity was observed for the prevalence of heart disease (OR = 4.80, I^2^ = 1%). For the influencing factors with a statistically significant heterogeneity, both fixed-effects and random-effects were used to combine the effect sizes. The results demonstrated a high degree of consistency between the calculations of the two models, affirming the reliability of this study outcomes.

Sepsis, blood transfusion, oral probiotics, and breastfeeding, which are the influencing factors involved in ≥ 10 publications, were separately funnel plotted. The results indicated asymmetry in the funnel plots (S Figs. 19-22). Egger’s and Begg’s test revealed publication bias (*P* < 0.05) for blood transfusion, oral probiotics, ICP, and mechanical ventilation (Table 3). Therefore, caution should be taken about the accuracy of the results.

**Table 3:** Sensitivity analysis and publication bias test

Influencing factors	Sensitivity analysis		Egger^,^ s test	
	Fixed effect model *OR (95% CI)*	Random effect model *OR (95% CI)*	* t* value	* P* value
Septicemia	3.91 (3.37, 4.55)	4.26 (3.41, 5.32)	2.52	0.019
Blood transfusion	2.41 (1.97, 2.95)	2.79 (2.04, 3.83)	2.56	0.027
Neonatal asphyxia	2.46 (2.07, 2.93)	2.81 (2.16, 3.67)	0.33	0.750
Pneumonia	6.17 (3.98, 9.57)	6.17 (3.98, 9.57)	-2.04	0.111
Infection occurs	5.99 (2.57, 9.74)	6.71 (2.14, 21.00)	-	-
Oral probiotics	0.70 (0.64, 0.77)	0.36 (0.25, 0.53)	-4.07	0.001
Breastfeeding	0.49 (0.45, 0.55)	0.31 (0.16, 0.62)	-0.74	0.477
Congenital heart disease	4.80 (3.00, 7.68)	4.80 (2.99, 7.70)	0.04	0.974
Intrahepatic cholestasis of pregnancy	2.71 (1.92, 3.82)	2.71 (1.92, 3.82)	6.05	0.026
Mechanical ventilation	1.44 (1.22, 1.71)	1.44 (1.22, 1.71)	16.57	0.004
Gestational diabetes	3.08 (1.73, 5.48)	3.08 (1.73, 5.48)	-	-
Respiratory distress syndrome	3.28 (2.23, 4.85)	3.28 (2.23, 4.85)	0.60	0.611
Prenatal application of glucocorticoids	0.38 (0.24, 0.60)	0.35 (0.20, 0.63)	-2.10	0.126
Hypoalbuminemia	2.80 (1.78, 4.41)	2.80 (1.78, 4.41)	-	-
Patent ductus arteriosus	3.10 (1.93, 4.98)	3.10 (1.93, 4.98)	1.4	0.394
respiratory failure	6.48 (2.92, 14.41)	7.51 (1.60, 35.10)	-	-
Severe anemia	2.86 (2.06, 3.99)	2.86 (2.06, 3.99)	2.07	0.175
History of antibiotic use	2.07 (1.57, 2.72)	2.12 (1.18, 3.81)	-	-
Meconium-stained amniotic fluid	3.14 (1.64, 6.01)	3.14 (1.64, 6.01)	-	-

## Discussion

To our knowledge, this is the first meta-analysis of factors influencing NEC in preterm infants in China to obtain an updated and thorough quantitative analysis. NEC has a rapid onset and progression of disease, with a high morbidity and mortality rate, and is one of the most important factors contributing to preterm infant mortality [64]. Exploring the protective and risk factors for NEC in preterm infants is important for prevention and developing effective interventions to reduce its incidence.

### Risk factors for NEC

This meta-analysis showed that MSAF, history of antibiotic use and preterm infection were risk factors for NEC in preterm infants. Chen et al. [45, 54, 63] also found that MSAF was an independent risk factor for NEC in preterm infants. The inhalation of amniotic fluid contaminated with meconium in utero in preterm infants can lead to the multiplication of intestinal pathogens and early infection [65]. In addition, due to the underdevelopment of the gastrointestinal tract, the imperfect barrier function of the intestinal mucosa, and the high permeability of the intestinal wall in preterm infants, bacteria can easily enter the intestinal tract and cause infections. The application of antibiotics affects the distribution of intestinal flora, leading to an increase in bacteria with potential therapeutic effects and a decrease in normal flora, which can damage the intestinal mucosal epithelium and lead to NEC [66]. The use of empirical antibiotics and the duration of antibiotic exposure in infants are associated with an increased risk of NEC [67]. In a meta-analysis of observational studies and randomized controlled trials, prophylactic antibiotic use in infants was not found to be statistically associated with NEC, but with an increased risk [68]. These factors influencing NEC are independent of each other while also influencing and interacting with one other. We also found sepsis to be a risk factor for NEC in preterm infants, consistent with the findings of Gagliardi et al. [69]. Comparing domestic and international studies on sepsis and NEC, we found that the OR values of sepsis for NEC reported abroad were mostly above 10 [70], while the OR values reported in China were mostly 3 to 5 [71], and the OR value of our study was 3.91. The fact that there are such obvious differences in such a strong risk factor as sepsis suggests that we should be cautious in interpreting the results of epidemiologic studies and medical statistics.

There is controversy surrounding whether blood transfusion in preterm infants contributes to an increased incidence of NEC. The present meta-analysis showed that blood transfusion is a risk factor for complications of NEC in preterm infants, while a recent meta-analysis showed that red blood cell (RBC) transfusion does not increase the risk of NEC [72]. Blood transfusions, especially large amounts of RBC, can cause impaired regulation of the mesenteric vessels, leading to increased adhesion and aggregation of RBC and formation of thrombi, resulting in poor blood flow to the intestine [73]. Whereas, a meta-analysis conducted by Rai et al. [74] demonstrated that erythrocyte infusion within 48 h exerted a protective effect on infants with NEC. Erythropoietin may have a protective effect on the endothelial cell barrier and therefore may attenuate the development of NEC. This study also found that severe anemia in preterm infants was associated with an increased risk of developing NEC [75]. Analysis of the reasons for this may be related to the fact that anemia decreases the expression of the tight junction protein ZO-1, increases the permeability of the intestinal barrier, increases intestinal inflammation by altering the function of macrophages, and predisposes patients to NEC. Patel et al. [75] reported that severe anemia, rather than RBC transfusion, was associated with an elevated risk of NEC and suggested that prevention of anemia may be more beneficial than minimizing RBC transfusions.

In this study, RDS, respiratory failure, neonatal asphyxia, and mechanical ventilation were all risk factors for NEC in preterm infants. Preterm combined VLBW infants are highly susceptible to asphyxia after birth due to their underdeveloped respiratory and neurological systems and their inability to perform effective gas exchange on their own. Infant asphyxia can induce the body’s defense reflex and redistribute the blood flow in the body to ensure the oxygen supply to the heart, brain, kidneys and other important organs, triggering strong constriction of mesenteric vessels, leading to ischemia and hypoxia of intestinal epithelial cells and even degeneration and necrosis, resulting in NEC [76]. Neonates with severe asphyxia often require mechanical ventilation, which was shown to be an independent risk factor for NEC in this study, and this is in agreement with the finding by Gagliardi et al. [69]. However, in a Canadian study, mechanical ventilation was shown to be a risk factor for NEC only in sicker infants [77].

This meta-analysis confirms that PDA, congenital heart disease, hypoproteinemia, and pneumonia are all risk factors for NEC in preterm infants. Preterm birth in combination with PDA and congenital heart disease causes inadequate intestinal blood flow, which induces immune responses, inflammatory mediators, and consequently intestinal mucosal injury and necrosis, leading to NEC [78]. At present, it is difficult to determine whether the presence of PDA or treatment with PDA alters the risk of NEC in infants. In a cohort of infants with PDA aged < 34 weeks, infants with PDA who were not treated with indomethacin had an increased risk of NEC compared with those who were treated [79]. However, in a recent meta-analysis of randomized controlled trials [80], it was found that there was no increased risk of NEC in the absence of PDA treatment.

In this study, maternal ICP and GDM were also found to be risk factors for NEC in preterm infants, which is consistent with the meta-analysis of Lu et al. [16]. Beghetti et al. [81] concluded that high concentrations of bile acids lead to enhanced contraction of placental villi veins exposed to amniotic fluid, reduced blood supply, and impaired intestinal microcirculation, resulting in intestinal mucosal ischemia and hypoxia and the production of multiple free radicals, forming the pathological basis for NEC. In GDM patients, the body is in a hyperglycemic state, and the fetus receives nutrients directly from the mother, which can affect the state of intestinal blood flow, lead to intestinal mucosal damage and induce NEC [30]. In this study, the pooled OR of ICP for NEC was 2.71 with four studies included; the pooled OR for GDM and NEC was 3.08 with only two included studies. Currently, there are fewer studies exploring the relationship between NEC and ICP, GDM, thus caution needs to be taken when making conclusion for the impact of GDM, ICP on NEC based on the observation in this study.

### Protect factors for NEC

The safety of oral probiotics in preterm infants is currently controversial, and it is believed that the optimal strain, optimal dose and duration of probiotics for the prevention of NEC have not been determined [82]. Our study found that probiotics reduced the risk of NEC, which is consistent with previously published meta-analysis [83]. Prophylactic supplementation with probiotics increases the deposition and growth of normal flora, enhances the barrier function of the intestinal mucosa, prevents the migration of bacteria or curative factors, and activates protective receptors to balance the intestinal flora [84]. The 2020 European Society of Pediatric Gastroenterology and Hepatology and Nutrition recommends that probiotics such as Bifidobacterium bifidum, when safe to do so, can be given to preterm infants to reduce the risk of NEC in preterm infants [85]. A multicenter randomized controlled study found that early administration of Bifidobacterium bifidum (BBG-001) did not reduce the risk of NEC in preterm infants [86]. The use of probiotics for the prevention of NEC in preterm infants is controversial and further studies are needed to demonstrate the effect.

In this meta-analysis, prenatal application of glucocorticoids was a protective factor for NEC in preterm infants, which is consistent with the results of a previous meta-analysis [17]. A Cochrane systematic evaluation documented that antenatal glucocorticoid use in women at risk of preterm labor reduced the incidence of RDS in preterm infants and also reduced the risk of NEC [87]. It was found that the proinflammatory factors interleukin-1β and interleukin-8 play an important role in the pathogenesis of NEC, and glucocorticoids significantly inhibit the proinflammatory effects of both and promote the maturation of the gastrointestinal tract while reducing the absorption of macromolecules by the intestinal mucosa and avoiding intestinal necrosis [88]. The clinical guidelines for the management of neonatal necrotizing small bowel colitis, published in China in 2020, recommend that glucocorticoids should be applied prenatally in mothers at risk of preterm delivery [89].

Breastfeeding has been shown to reduce the risk of NEC. In this study, breastfeeding was a protective factor for NEC in preterm infants, and a review by Nolan et al. [90] indicated that immune components in breast milk have a protective effect against NEC. A prospective study found that breastfeeding reduced the risk of NEC in preterm infants compared with formula feeding [91]. Breast milk, with its lower osmolality compared to formula, alleviates the osmotic load of food, relieving intestinal pressure. In addition, breast milk is rich in secretory IgA, lactoferrin and other antimicrobial active substances, which enhance the body’s immune defense and effectively prevent the occurrence of infectious diseases of the gastrointestinal tract [92].

### Limitations

Our study had several limitations. First, we only included published literature, and potential publication bias should not be ignored. Second, the quality of the included articles was limited and the heterogeneity among studies was high, and we need to be cautious in interpreting the findings. We only included NEC premature infants hospitalized in neonatal intensive care units (NICUs) in China, and there was some bias in the selection of the study population and region, which may limit the generalizability of the findings. In addition, this study also found that cesarean section and the use of pulmonary surfactant are protective factors against NEC in premature infants, but due to the limited number of included studies, meta-analysis was not performed on these factors. Although this study only analyzed the factors influencing NEC in Chinese preterm infants, it may provide a valuable foundation for future research and interventions to enhance infants’ health related to NEC.

## Conclusion

In summary, our study suggests that septicemia, blood transfusion, neonatal asphyxia, pneumonia, infection, congenital heart disease, ICP, GDM, RDS, hypoproteinemia, PDA, respiratory failure, severe anemia, antibiotic use history and MSAF are risk factors for NEC in premature infants in China. Breastfeeding, oral probiotics and prenatal use of glucocorticoids are protective factors. This is the first meta-analysis of the factors influencing NEC in preterm infants in China, further expanding our knowledge in this subject area.

### Supplementary Information

**Additional file 1.**

**Additional file 2:** **Supplementary Information Table 2.** Text database search criteria.

** Additional file 3.** AMSTAR 2: a critical appraisal tool for systematic reviews that include randomised or non- randomised studies of healthcare interventions, or both

** Additional file 4: Table S3.** Assessment of methodological quality by NOS.

** Additional file 5: S figure1.** Forest plot of the analysis regarding blood transfusion as a risk factor for NEC preterm infants. **S figure2.** Forest plot of the analysis regarding neonatal asphyxia as a risk factor for NEC preterm infants. **S figure 3.** Forest plot of the analysis regarding pneumonia as a risk factor for NEC preterm infants. **S figure 4.** Forest plot of the analysis regarding infection occurs as a risk factor for NEC preterm infants. **S figure 5.** Forest plot of the analysis regarding breastfeeding as a protective factor for NEC preterm infants. **S figure 6.** Forest plot of the analysis regarding congenital heart disease as a risk factor for NEC preterm infants. **S figure 7.** Forest plot of the analysis regarding meconium-stained amniotic fluid as a risk factor for NEC preterm infants. **S figure 8.** Forest plot of the analysis regarding mechanical ventilation as a risk factor for NEC preterm infants. **S figure 9.** Forest plot of the analysis regarding gestational diabetes mellitus as a risk factor for NEC preterm infants. **S figure 10.** Forest plot of the analysis regarding respiratory distress syndrome as a risk factor for NEC preterm infants. **S figure 11.** Forest plot of the analysis regarding prenatal application of glucocorticoids as a protective factor for NEC preterm infants. **S figure 12.** Forest plot depicting the analysis of intravenous immunoglobulin as a non-influencing factor for NEC preterm infants. **S figure 13.** Forest plot of the analysis regarding hypoalbuminemia as a risk factor for NEC preterm infants. **S figure 14.** Forest plot of the analysis regarding patent ductus arteriosus as a risk factor for NEC preterm infants. **S figure 15.** Forest plot of the analysis regarding respiratory failure as a risk factor for NEC preterm infants. **S figure 16.** Forest plot of the analysis regarding severe anemia as a risk factor for NEC preterm infants. **S figure 17.** Forest plot of the analysis regarding history of antibiotic use as a risk factor for NEC preterm infants. **S figure 18.** Forest plot of the analysis regarding intrahepatic cholestasis of pregnancy as a risk factor for NEC preterm infants.

** Additional file 6: S figure19.** Funnel plot of breastfeeding. **S figure20.** Funnel plot of blood transfusion. **S figure21.** Funnel plot of oral probiotics. **S figure22.** Funnel plot of septicemia.

## Data Availability

The datasets used and/or analyzed during the current study are available from the corresponding author on reasonable request.

## Abbreviations

ANS: Antenatal steroid
CI: Confidence interval
CNKI: China National Knowledge Infrastructure
CBM: China Biomedical Literature Database
IVIG: Intravenous Immunoglobulin
ICP: Intrahepatic cholestasis of pregnancy
MSAF: Meconium-stained amniotic fluid
NEC: Necrotizing enterocolitis
NOS: Newcastle-Ottawa Scale
NICU: Neonatal intensive care unit
OR: Odds ratio
PDA: Patent ductus arteriosus
RDS: Respiratory distress syndrome
GDM: Gestational diabetes mellitus
PRISMA: Preferred Reporting Items for Systematic Reviews and Meta-Analyses
AMSTAR2: A Measurement Tool to Assess Systematic Reviews 2
VLBW: Very low birth weight
WHO: World health organization
GA: Gestational age
BW: Birth weight
