# Supplementary material for: Factors influencing necrotizing enterocolitis in premature infants in China: a systematic review and meta-analysis
Source: BMC Pediatr. 2024 Feb 29;24:148. doi: 10.1186/s12887-024-04607-3 (PMC10903018; doi:10.1186/s12887-024-04607-3)
Supplement: Supplementary file 2 — Additional file 2: Supplementary Information Table 2. Text database search criteria. [file 12887_2024_4607_MOESM2_ESM.docx]

**Supplementary Information Table 2 Text database** **search criteria**

1. **Search criteria for PubMed:**

**#1** "Infant, Premature"[MeSH Terms] OR "Prematurity" OR "preterm infant "OR "Neonatal Prematurity" OR "Extremely Premature Infant" OR "Extremely Preterm Infant"[All Fields]

**#2** "Risk Factors"[MeSH Terms] OR "relative risk "OR "dangerous factors" OR "relevant factors" OR "influence factors" OR "influencing factor"[All Fields]

**#3** "Enterocolitis, Necrotizing"[MeSH Terms] OR" Necrotizing Enterocolitis" OR "acute necrotising enterocolitis" OR" enterocolitis, acute necrotising "OR "enterocolitis, acute necrotizing "OR "enterocolitis, necrotising" OR" necrotising enterocolitis"[All Fields]

**#4** "China"[MeSH Terms] OR "Chinese"[All Fields]

**#5** #1 AND #2 AND #3 AND #4 **175**

1. **Search criteria for Embase:**

**#1** 'prematurity'/exp OR 'infant, premature':ab,ti OR prematurity:ab,ti OR 'preterm infant':ab,ti OR 'neonatal prematurityor extremely premature infant':ab,ti OR 'extremely preterm infant':ab,ti

**#2** 'risk factor'/exp OR 'relative risk':ab,ti OR 'dangerous factors':ab,ti OR 'relevant factors':ab,ti OR 'influence factors':ab,ti OR 'influencing factor':ab,ti

**#3** 'necrotizing enterocolitis'/exp OR 'enterocolitis, necrotizing':ab,ti OR 'acute necrotising enterocolitis':ab,ti OR 'enterocolitis, acute necrotising':ab,ti OR 'enterocolitis, acute necrotizing':ab,ti OR 'enterocolitis, necrotising':ab,ti OR 'necrotising enterocolitis':ab,ti

**#4** chinese OR china

**#5** #1 AND #2 AND #3 AND #4  **97**

**C) Search criteria for Web of Science:**

**#1** TS= (" Infant, Premature" OR "Prematurity" OR "preterm infant "OR "Neonatal Prematurity" OR "Extremely Premature Infant" OR "Extremely Preterm Infant")

**#2** TS= ("Risk Factors"OR "relative risk "OR "dangerous factors" OR "relevant factors" OR "influence factors" OR "influencing factor")

**#3** TS= ("Enterocolitis, Necrotizing"OR" Necrotizing Enterocolitis" OR "acute necrotising enterocolitis" OR" enterocolitis, acute necrotising "OR "enterocolitis, acute necrotizing "OR "enterocolitis, necrotising" OR" necrotising enterocolitis")

**#4** #1 AND #2 AND #3  **305**

**D) Search criteria for the Cochrane Library:**

**#1** (Prematurity OR preterm infant OR Neonatal Prematurity OR Extremely Premature Infant OR Extremely Preterm Infant OR "Infant, Premature"):ti,ab,kw

**#2**(Necrotizing Enterocolitis OR acute necrotising enterocolitis OR acute necrotizing enterocolitis OR enterocolitis necroticans OR enterocolitis, acute necrotising OR enterocolitis, acute necrotizing OR enterocolitis, necrotising OR enterocolitis, necrotizing OR necrotising enterocolitis OR "Enterocolitis, Necrotizing"):ti,ab,kw

**#3** (relative risk OR dangerous factors OR relevant factors or influence factors or influencing factor or "Risk Factors"):ti,ab,kw

#4 #1AND #2 AND #3  **262**

**E) Search criteria for CNKI:**

**#1** "Infant, Premature"OR "Prematurity" OR "preterm infant "OR "Neonatal Prematurity" OR "Extremely Premature Infant" OR "Extremely Preterm Infant"

**#2** "Risk Factors" OR "relative risk "OR "dangerous factors" OR "relevant factors" OR "influence factors" OR "influencing factor"

**#3** "Enterocolitis, Necrotizing"OR" Necrotizing Enterocolitis" OR "acute necrotising enterocolitis" OR" enterocolitis, acute necrotising "OR "enterocolitis, acute necrotizing "OR "enterocolitis, necrotising" OR" necrotising enterocolitis"

**#4** #1 AND #2 AND #3  **281**

**F) Search criteria for Wanfang :**

**#1** "Infant, Premature"OR "Prematurity" OR "preterm infant "OR "Neonatal Prematurity" OR "Extremely Premature Infant" OR "Extremely Preterm Infant"

**#2** "Risk Factors" OR "relative risk "OR "dangerous factors" OR "relevant factors" OR "influence factors" OR "influencing factor"

**#3** "Enterocolitis, Necrotizing"OR" Necrotizing Enterocolitis" OR "acute necrotising enterocolitis" OR" enterocolitis, acute necrotising "OR "enterocolitis, acute necrotizing "OR "enterocolitis, necrotising" OR" necrotising enterocolitis"

**#4** #1 AND #2 AND #3  **690**

**G) Search criteria for VIP:**

**#1** "Infant, Premature"OR "Prematurity" OR "preterm infant "OR "Neonatal Prematurity" OR "Extremely Premature Infant" OR "Extremely Preterm Infant"

**#2** "Risk Factors" OR "relative risk "OR "dangerous factors" OR "relevant factors" OR "influence factors" OR "influencing factor"

**#3** "Enterocolitis, Necrotizing"OR" Necrotizing Enterocolitis" OR "acute necrotising enterocolitis" OR" enterocolitis, acute necrotising "OR "enterocolitis, acute necrotizing "OR "enterocolitis, necrotising" OR" necrotising enterocolitis"

**#4** #1 AND #2 AND #3  **377**

**H) Search criteria for SinoMed:**

**#1** "Infant, Premature"OR "Prematurity" OR "preterm infant "OR "Neonatal Prematurity" OR "Extremely Premature Infant" OR "Extremely Preterm Infant"

**#2** "Risk Factors" OR "relative risk "OR "dangerous factors" OR "relevant factors" OR "influence factors" OR "influencing factor"

**#3** "Enterocolitis, Necrotizing"OR" Necrotizing Enterocolitis" OR "acute necrotising enterocolitis" OR" enterocolitis, acute necrotising "OR "enterocolitis, acute necrotizing "OR "enterocolitis, necrotising" OR" necrotising enterocolitis"

**#4** #1 AND #2 AND #3  **303**
