# Supplementary material for: Factors influencing necrotizing enterocolitis in premature infants in China: a systematic review and meta-analysis
Source: BMC Pediatr. 2024 Feb 29;24:148. doi: 10.1186/s12887-024-04607-3 (PMC10903018; doi:10.1186/s12887-024-04607-3)
Supplement: Supplementary file 4 — Additional file 4: Table S3. Assessment of methodological quality by NOS. [file 12887_2024_4607_MOESM4_ESM.docx]

Table S3. Assessment of methodological quality by NOS

| Study | Selection | | | | Comparability | Exposure | | | Total |
| --- | --- | --- | --- | --- | --- | --- | --- | --- | --- |
|  | Is the case definition adequate | Representativeness of the cases | Selection of Controls | Definition of Controls | Comparability of cases and controls on the basis of the design or analysis | Ascertainment of exposure | Same method of ascertainment for cases and controls | Non-Response rate |  |
| Zeng SY 2021 | 1 | 1 | 0 | 1 | 2 | 1 | 1 | 0 | 7 |
| Cheng SP 2016 | 1 | 1 | 0 | 1 | 2 | 1 | 1 | 1 | 8 |
| Liu YC 2019 | 1 | 1 | 0 | 1 | 2 | 1 | 1 | 0 | 7 |
| Shang Y 2014 | 1 | 1 | 0 | 1 | 2 | 1 | 1 | 0 | 7 |
| Sun HY 2017 | 1 | 1 | 0 | 1 | 2 | 1 | 1 | 1 | 8 |
| Dong HM 2021 | 1 | 1 | 0 | 1 | 2 | 1 | 1 | 0 | 7 |
| Ru Y 2022 | 1 | 1 | 0 | 1 | 1 | 1 | 1 | 0 | 6 |
| Wang YP 2022 | 1 | 1 | 0 | 1 | 2 | 1 | 1 | 0 | 7 |
| Li XH 2019 | 1 | 1 | 0 | 0 | 2 | 1 | 1 | 0 | 6 |
| Ru H 2018 | 1 | 1 | 0 | 1 | 2 | 1 | 1 | 0 | 7 |
| Ru XY 2013 | 1 | 1 | 0 | 1 | 1 | 1 | 1 | 0 | 6 |
| Lu M 2015 | 1 | 1 | 0 | 1 | 1 | 1 | 1 | 0 | 6 |
| Qu XL 2019 | 1 | 1 | 0 | 1 | 2 | 1 | 1 | 1 | 8 |
| Shi Y 2019 | 1 | 1 | 0 | 1 | 2 | 1 | 1 | 0 | 7 |
| Wang RJ 2014 | 1 | 1 | 0 | 1 | 2 | 1 | 1 | 0 | 7 |
| Yu M 2018 | 1 | 1 | 0 | 1 | 2 | 1 | 1 | 0 | 7 |
| Zhu MY 2012 | 0 | 1 | 0 | 1 | 2 | 2 | 1 | 1 | 8 |
| Hou AN 2017 | 1 | 1 | 0 | 1 | 2 | 1 | 1 | 0 | 7 |
| Song J 2021 | 1 | 1 | 0 | 1 | 2 | 2 | 1 | 1 | 9 |
| Zhang LP 2017 | 1 | 1 | 0 | 1 | 2 | 2 | 1 | 0 | 8 |
| Ru Q 2017 | 1 | 1 | 0 | 1 | 2 | 2 | 1 | 1 | 9 |
| Zhu KL 2021 | 1 | 1 | 0 | 1 | 2 | 2 | 1 | 1 | 9 |
| Huang D 2022 | 1 | 1 | 0 | 1 | 2 | 1 | 1 | 0 | 7 |
| Liu X 2022 | 1 | 1 | 0 | 1 | 2 | 2 | 1 | 0 | 8 |
| Yang J 2022 | 1 | 1 | 0 | 1 | 2 | 1 | 1 | 1 | 8 |
| Yang LR 2018 | 1 | 1 | 0 | 1 | 2 | 1 | 1 | 0 | 7 |
| Zhuang XY 2007 | 0 | 1 | 0 | 1 | 2 | 1 | 1 | 0 | 6 |
| Li ZT 2020 | 1 | 1 | 0 | 1 | 2 | 1 | 1 | 0 | 7 |
| Ma XJ 2021 | 0 | 1 | 0 | 1 | 1 | 1 | 1 | 1 | 6 |
| Deng X 2017 | 1 | 1 | 0 | 1 | 1 | 1 | 1 | 0 | 6 |
| Zhu JL 2020 | 1 | 1 | 0 | 1 | 2 | 1 | 1 | 1 | 8 |
| Wang XQ 2017 | 1 | 1 | 0 | 1 | 1 | 1 | 1 | 1 | 7 |
| Wang PP 2020 | 0 | 1 | 0 | 1 | 2 | 1 | 1 | 0 | 6 |
| Zhang L 2017 | 0 | 1 | 0 | 1 | 1 | 1 | 1 | 1 | 6 |
| Chen S 2020 | 1 | 1 | 0 | 1 | 2 | 1 | 1 | 1 | 8 |
| Tan XR 2022 | 1 | 1 | 0 | 1 | 2 | 2 | 1 | 1 | 9 |
| Yu ZY 2023 | 1 | 1 | 0 | 1 | 2 | 2 | 1 | 1 | 9 |
| Tain M 2023 | 1 | 1 | 0 | 1 | 2 | 1 | 1 | 0 | 7 |
