# Supplementary material for: Factors influencing necrotizing enterocolitis in premature infants in China: a systematic review and meta-analysis
Source: BMC Pediatr. 2024 Feb 29;24:148. doi: 10.1186/s12887-024-04607-3 (PMC10903018; doi:10.1186/s12887-024-04607-3)
Supplement: Supplementary file 5 — Additional file 5: S figure1. Forest plot of the analysis regarding blood transfusion as a risk factor for NEC preterm infants. S figure2. Forest plot of the analysis regarding neonatal asphyxia as a risk factor for NEC preterm infants. S figure 3. Forest plot of the analysis regarding pneumonia as a risk factor for NEC preterm infants. S figure 4. Forest plot of the analysis regarding infection occurs as a risk factor for NEC preterm infants. S figure 5. Forest plot of the analysis regarding breastfeeding as a protective factor for NEC preterm infants. S figure 6. Forest plot of the analysis regarding congenital heart disease as a risk factor for NEC preterm infants. S figure 7. Forest plot of the analysis regarding meconium-stained amniotic fluid as a risk factor for NEC preterm infants. S figure 8. Forest plot of the analysis regarding mechanical ventilation as a risk factor for NEC preterm infants. S figure 9. Forest plot of the analysis regarding gestational diabetes mellitus as a risk factor for NEC preterm infants. S figure 10. Forest plot of the analysis regarding respiratory distress syndrome as a risk factor for NEC preterm infants. S figure 11. Forest plot of the analysis regarding prenatal application of glucocorticoids as a protective factor for NEC preterm infants. S figure 12. Forest plot depicting the analysis of intravenous immunoglobulin as a non-influencing factor for NEC preterm infants. S figure 13. Forest plot of the analysis regarding hypoalbuminemia as a risk factor for NEC preterm infants. S figure 14. Forest plot of the analysis regarding patent ductus arteriosus as a risk factor for NEC preterm infants. S figure 15. Forest plot of the analysis regarding respiratory failure as a risk factor for NEC preterm infants. S figure 16. Forest plot of the analysis regarding severe anemia as a risk factor for NEC preterm infants. S figure 17. Forest plot of the analysis regarding history of antibiotic use as a risk factor for NEC preterm infants. S f [file 12887_2024_4607_MOESM5_ESM.docx]

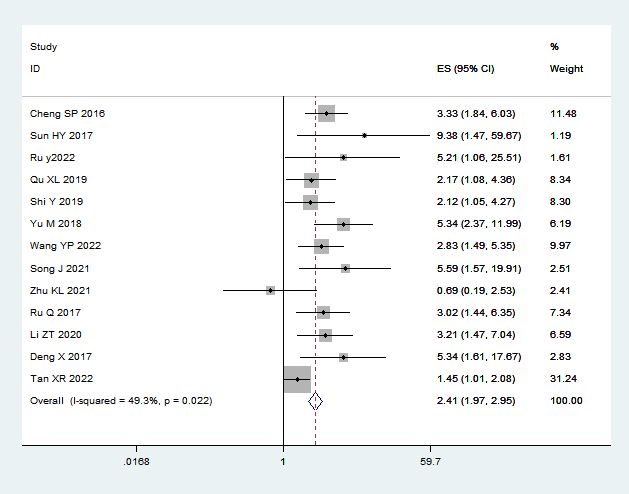


S figure1. Forest plot of the analysis regarding blood transfusion as a risk factor for NEC preterm infants. Note: NEC: necrotizing enterocolitis; ES: Odds ratio (OR).


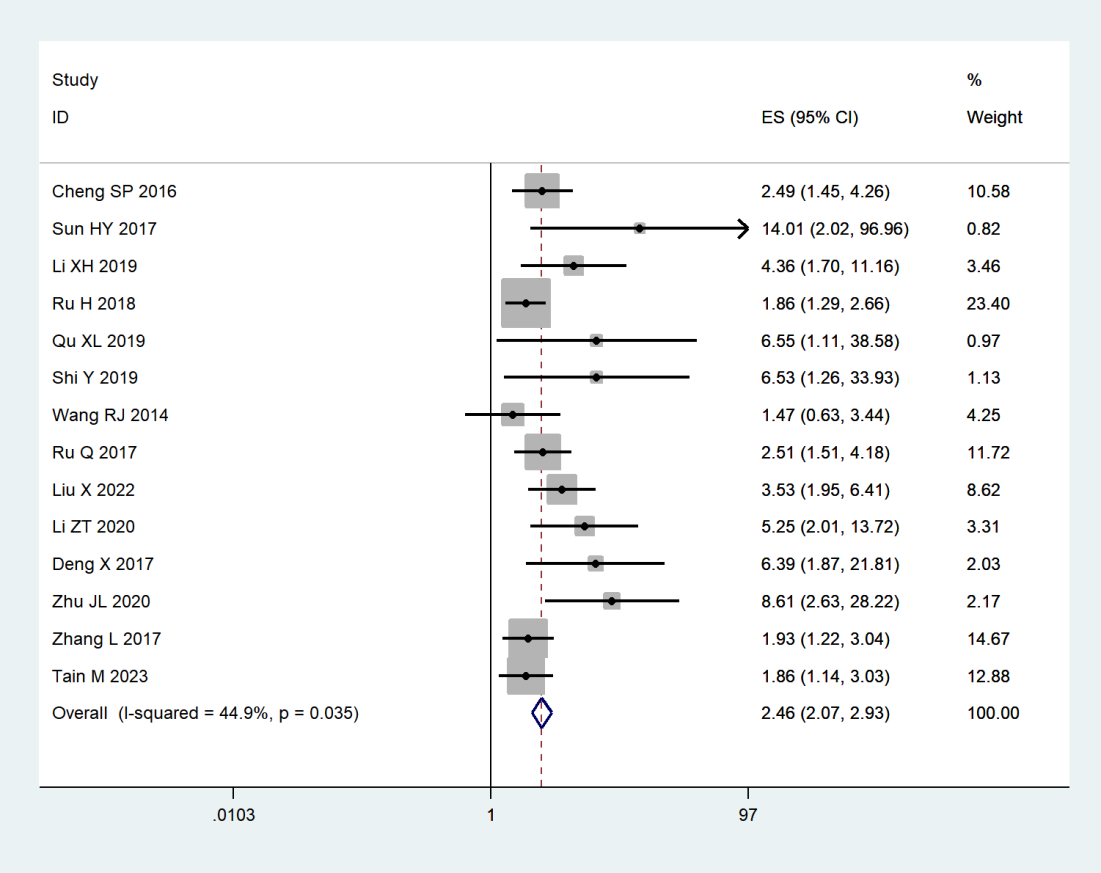


S figure2. Forest plot of the analysis regarding neonatal asphyxia as a risk factor for NEC preterm infants. Note: NEC: necrotizing enterocolitis; ES: Odds ratio (OR).


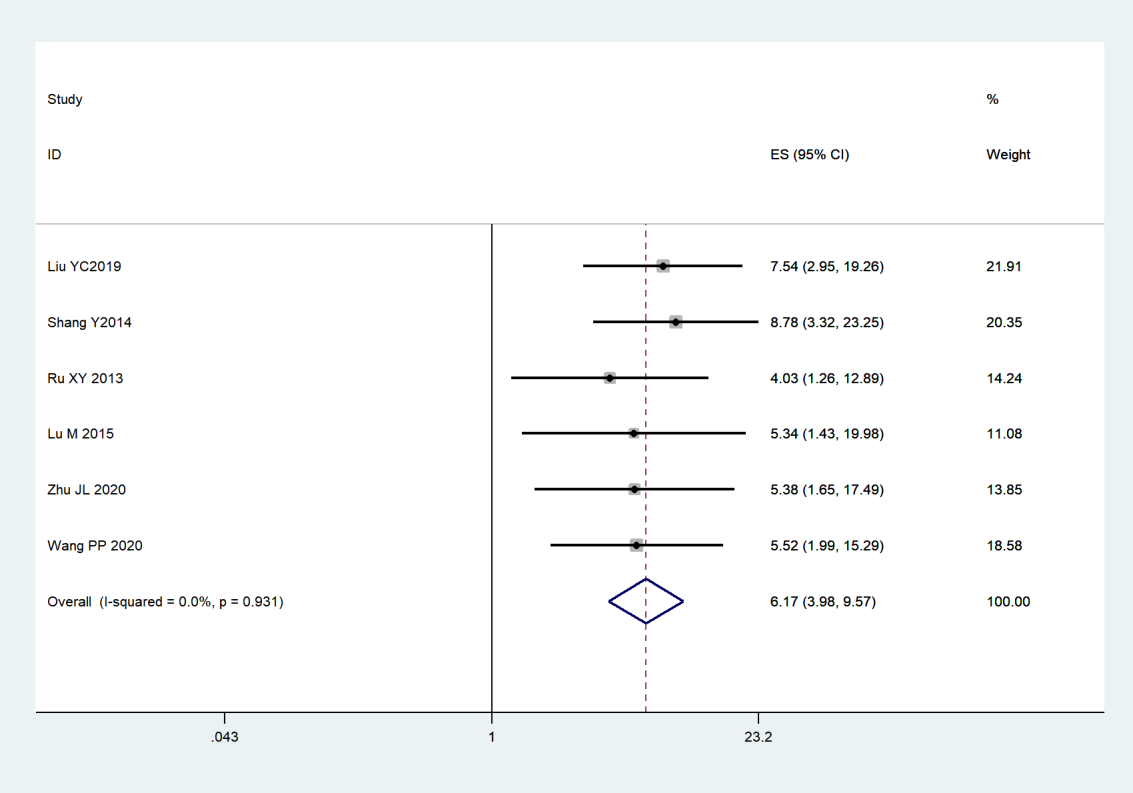


S figure 3. Forest plot of the analysis regarding pneumonia as a risk factor for NEC preterm infants. Note: NEC: necrotizing enterocolitis; ES: Odds ratio (OR).


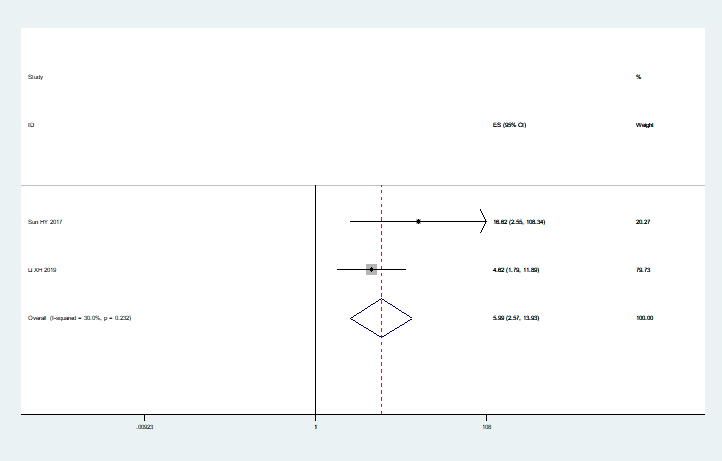


S figure 4. Forest plot of the analysis regarding infection occurs as a risk factor for NEC preterm infants. Note: NEC: necrotizing enterocolitis; ES: Odds ratio (OR).


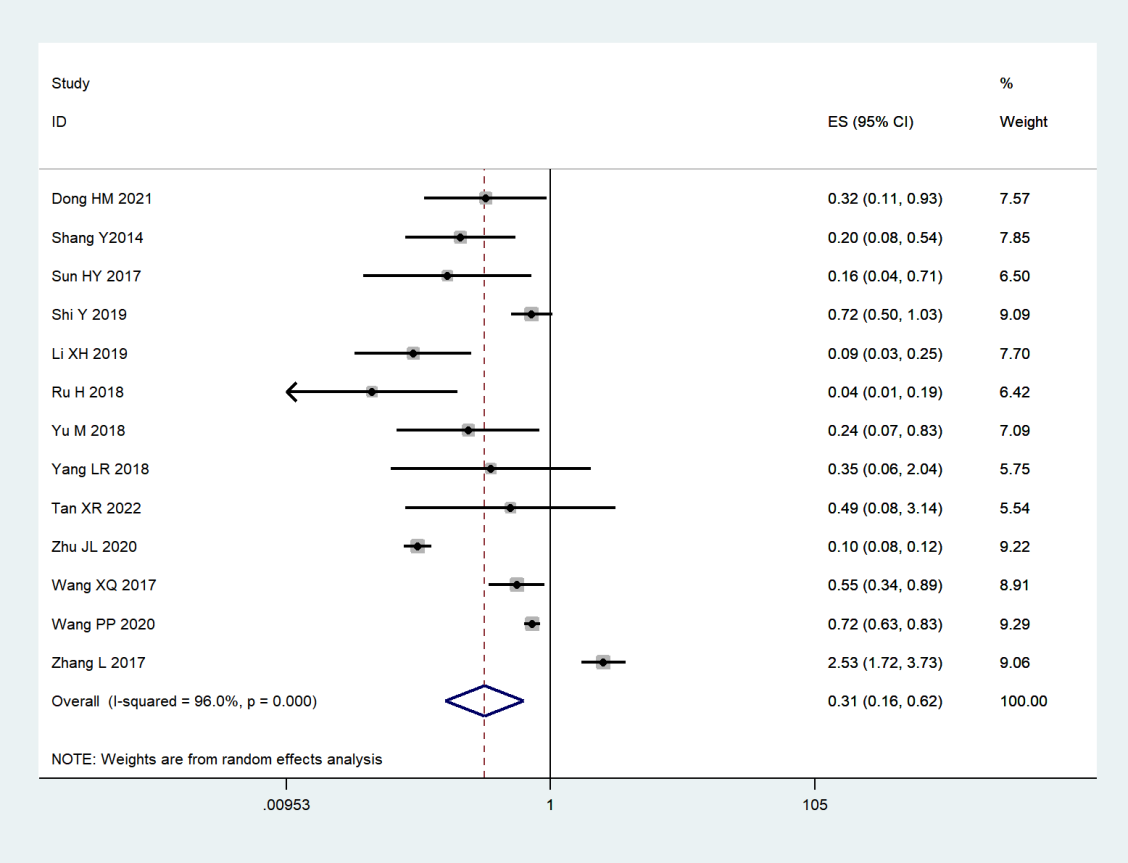


S figure 5. Forest plot of the analysis regarding breastfeeding as a protective factor for NEC preterm infants. Note: NEC: necrotizing enterocolitis; ES: Odds ratio (OR).


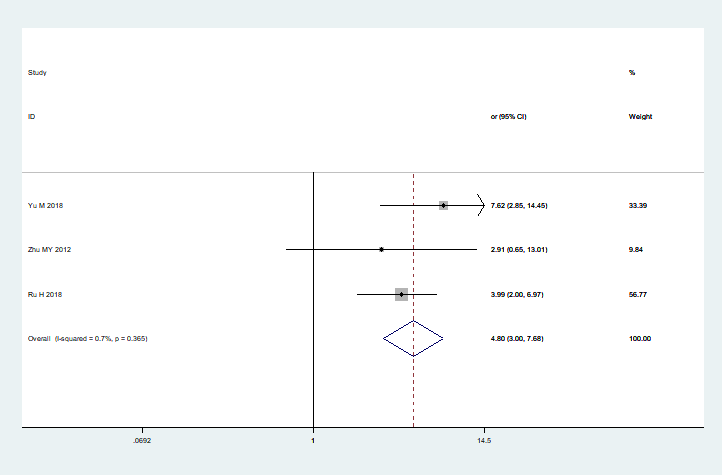


S figure 6. Forest plot of the analysis regarding congenital heart disease as a risk factor for NEC preterm infants. Note: NEC: necrotizing enterocolitis; ES: Odds ratio (OR).


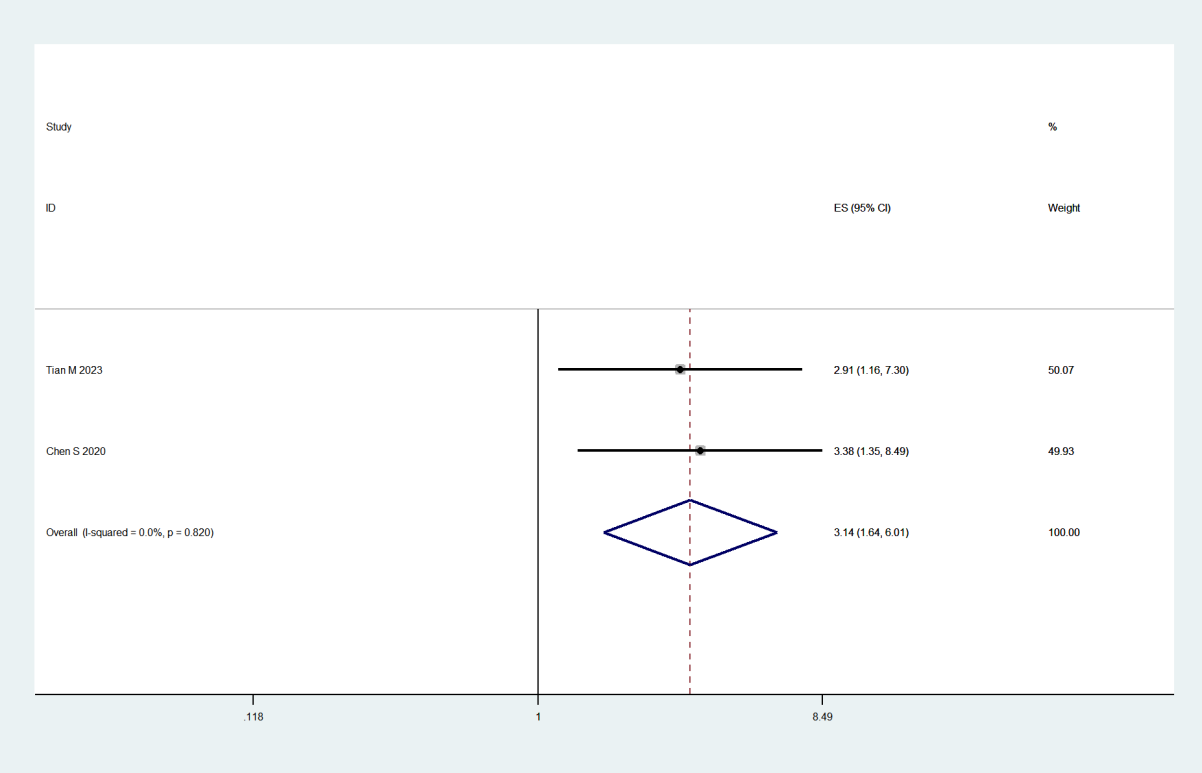


S figure 7. Forest plot of the analysis regarding meconium-stained amniotic fluid as a risk factor for NEC preterm infants. Note: NEC: necrotizing enterocolitis; ES: Odds ratio (OR).


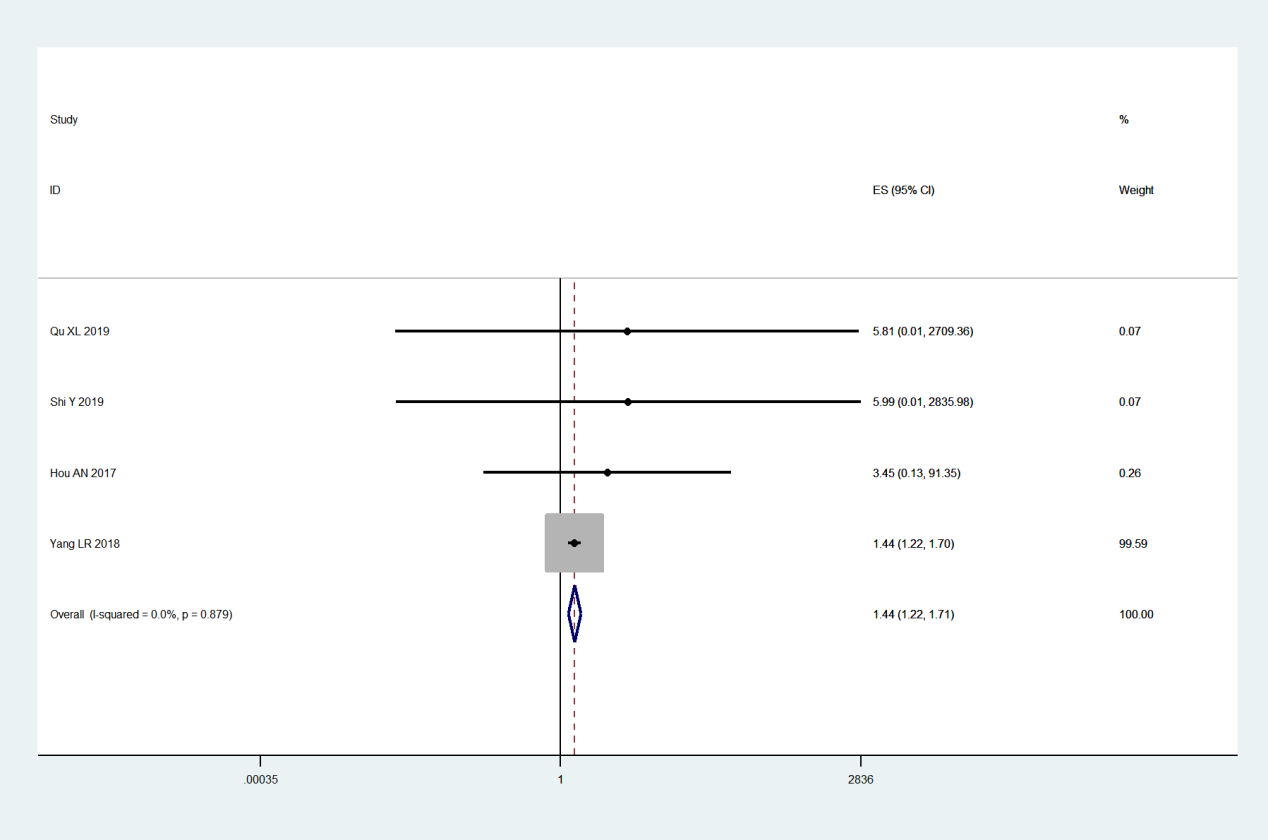


S figure 8. Forest plot of the analysis regarding mechanical ventilation as a risk factor for NEC preterm infants. Note: NEC: necrotizing enterocolitis; ES: Odds ratio (OR).


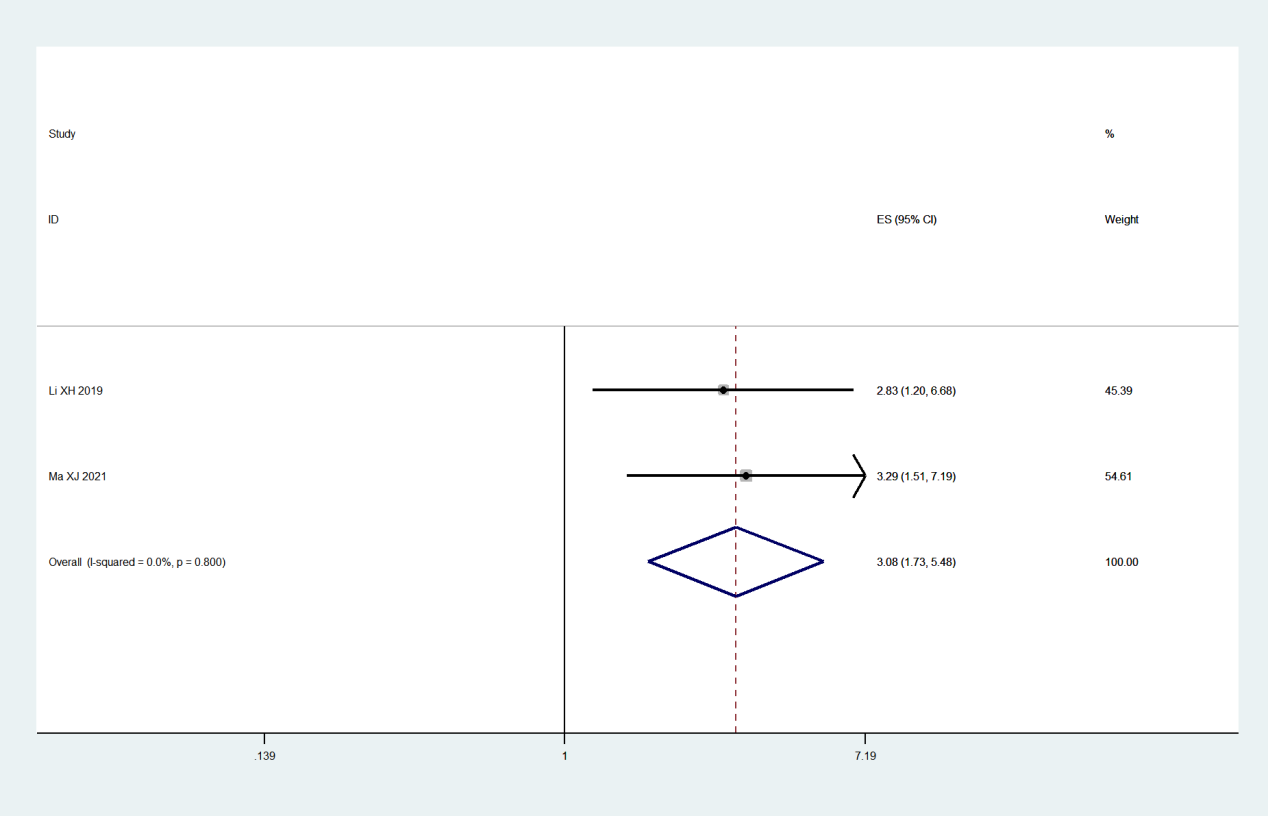


S figure 9. Forest plot of the analysis regarding gestational diabetes mellitus as a risk factor for NEC preterm infants. Note: NEC: necrotizing enterocolitis; ES: Odds ratio (OR).


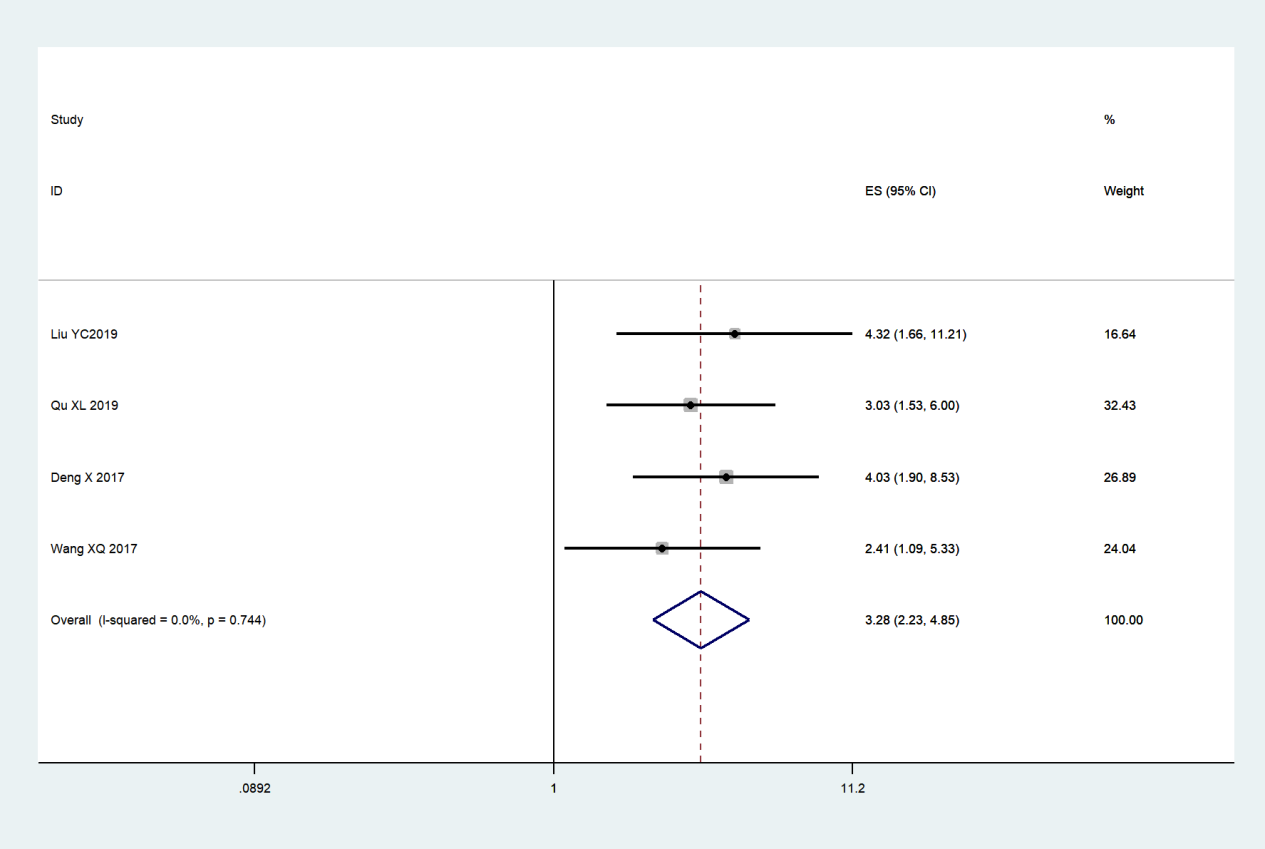


S figure 10. Forest plot of the analysis regarding respiratory distress syndrome as a risk factor for NEC preterm infants. Note: NEC: necrotizing enterocolitis; ES: Odds ratio (OR).


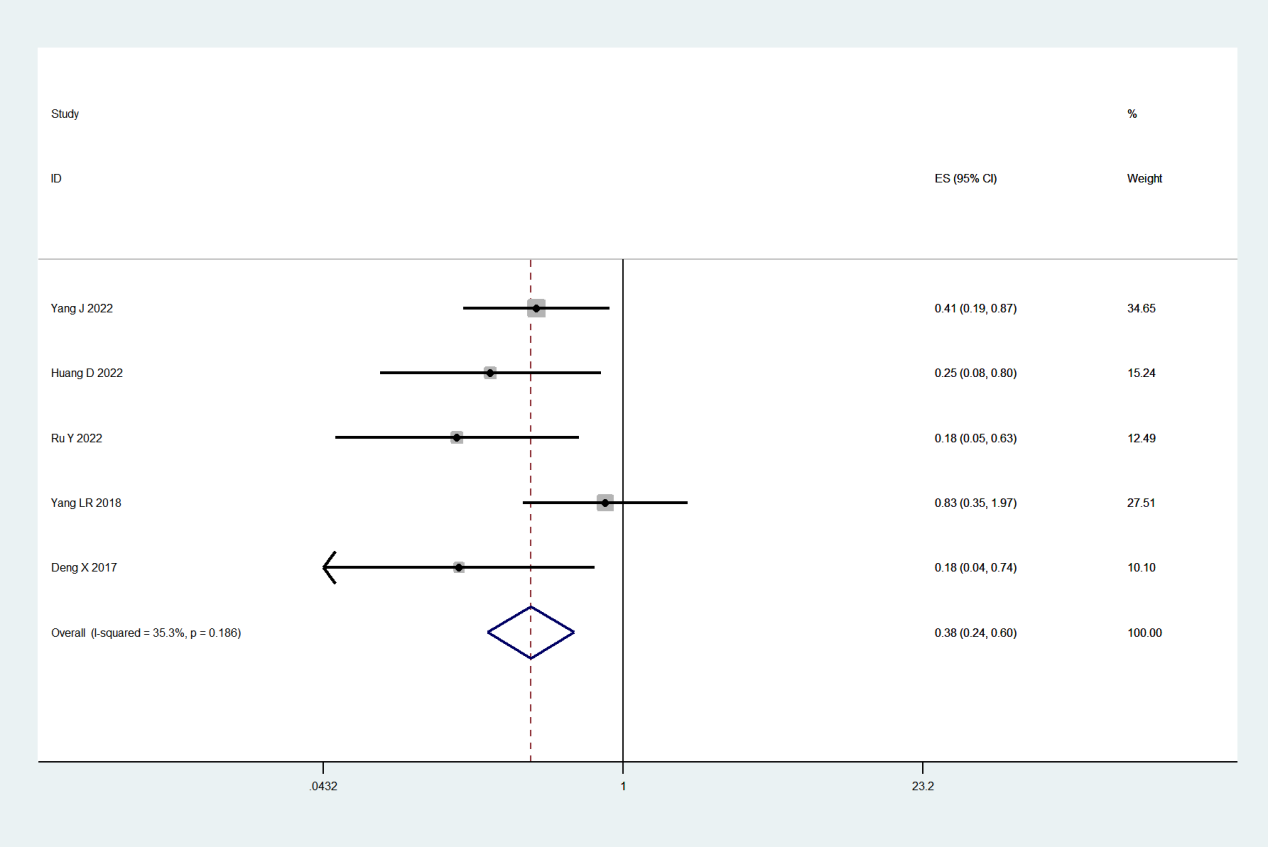


S figure 11. Forest plot of the analysis regarding prenatal application of glucocorticoids as a protective factor for NEC preterm infants. Note: NEC: necrotizing enterocolitis; ES: Odds ratio (OR).


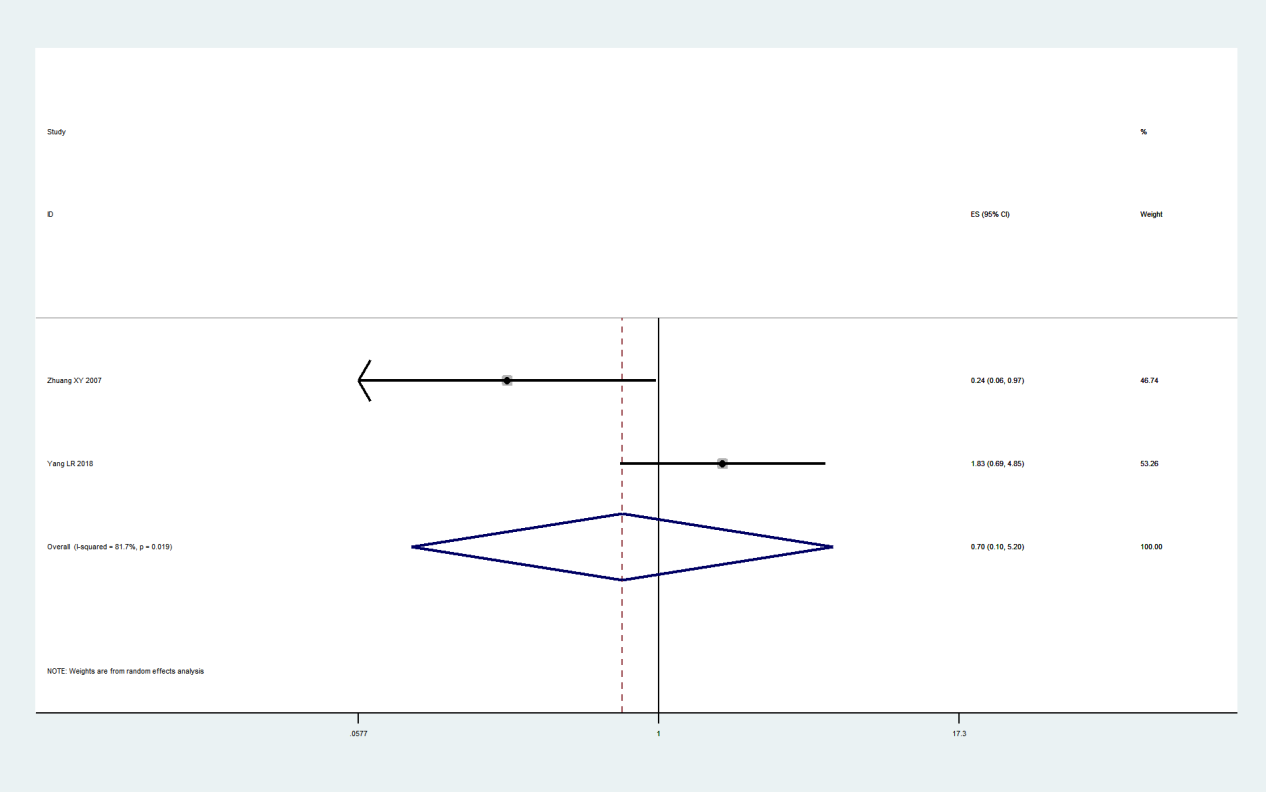


S figure 12. Forest plot depicting the analysis of intravenous immunoglobulin as a non-influencing factor for NEC preterm infants. Note: NEC: necrotizing enterocolitis; ES: Odds ratio (OR).


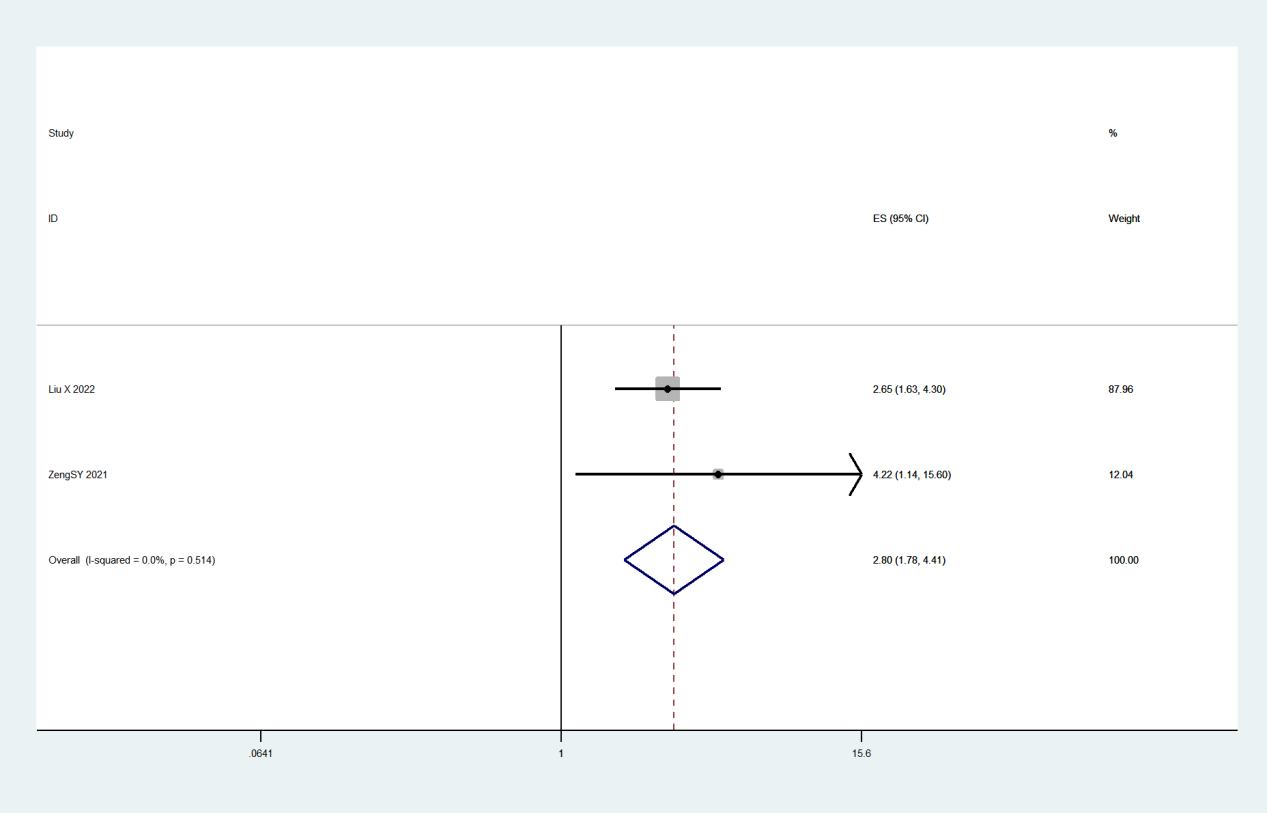


S figure 13. Forest plot of the analysis regarding hypoalbuminemia as a risk factor for NEC preterm infants. Note: NEC: necrotizing enterocolitis; ES: Odds ratio (OR).


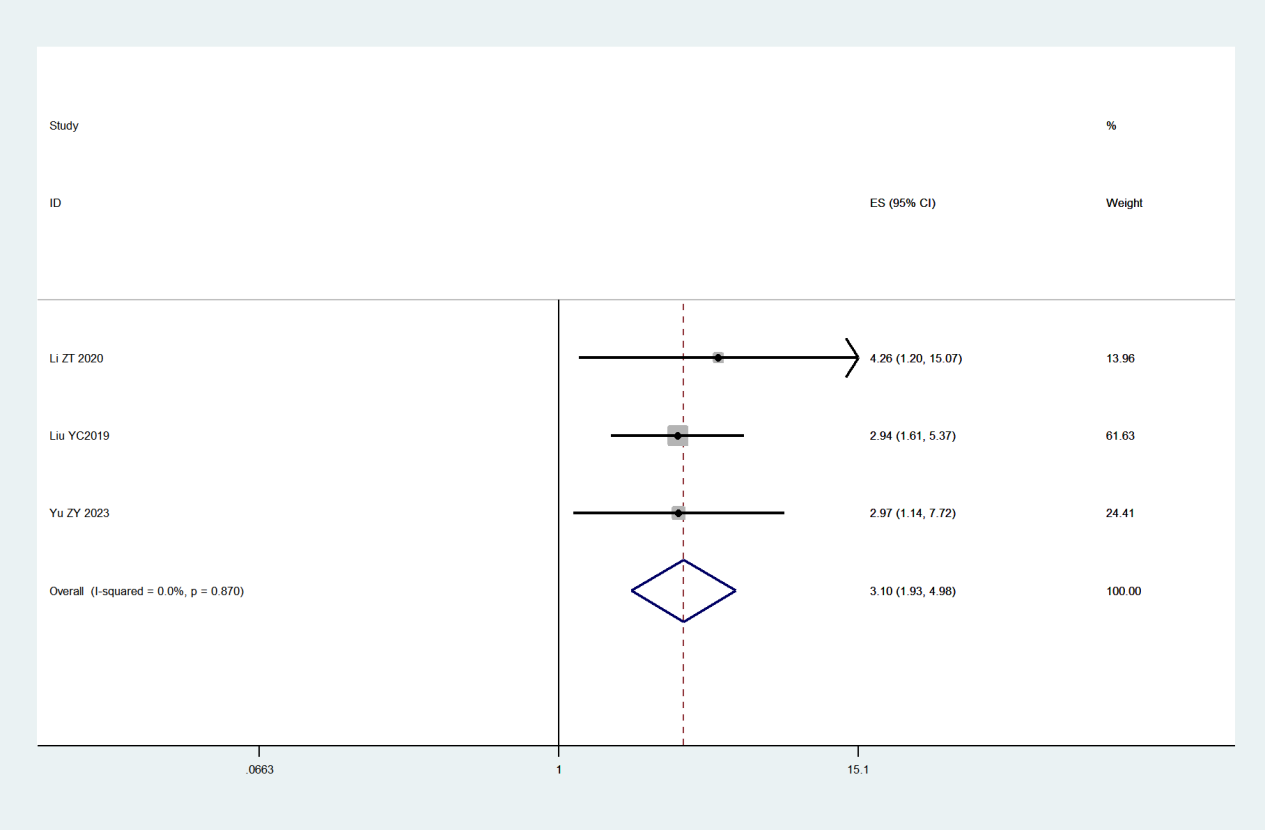


S figure 14. Forest plot of the analysis regarding patent ductus arteriosus as a risk factor for NEC preterm infants. Note: NEC: necrotizing enterocolitis; ES: Odds ratio (OR).


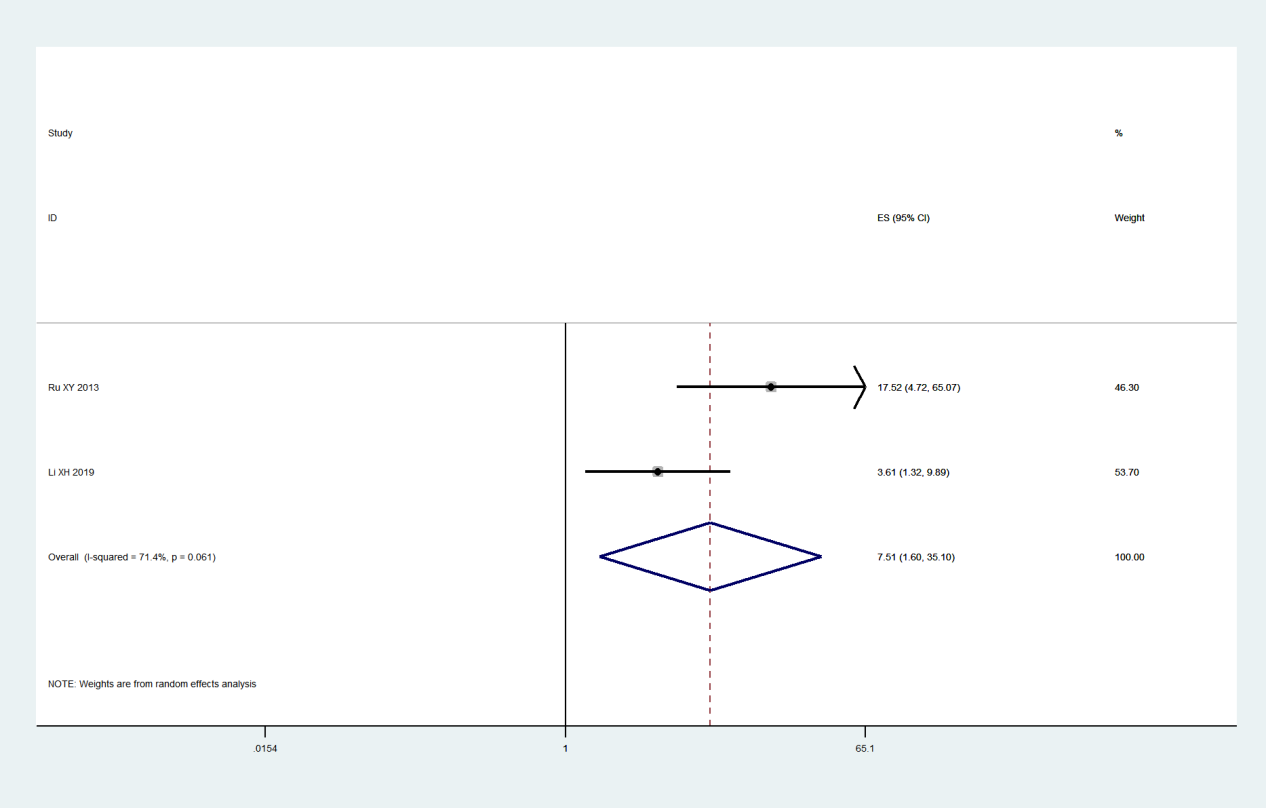


S figure 15. Forest plot of the analysis regarding respiratory failure as a risk factor for NEC preterm infants. Note: NEC: necrotizing enterocolitis; ES: Odds ratio (OR).


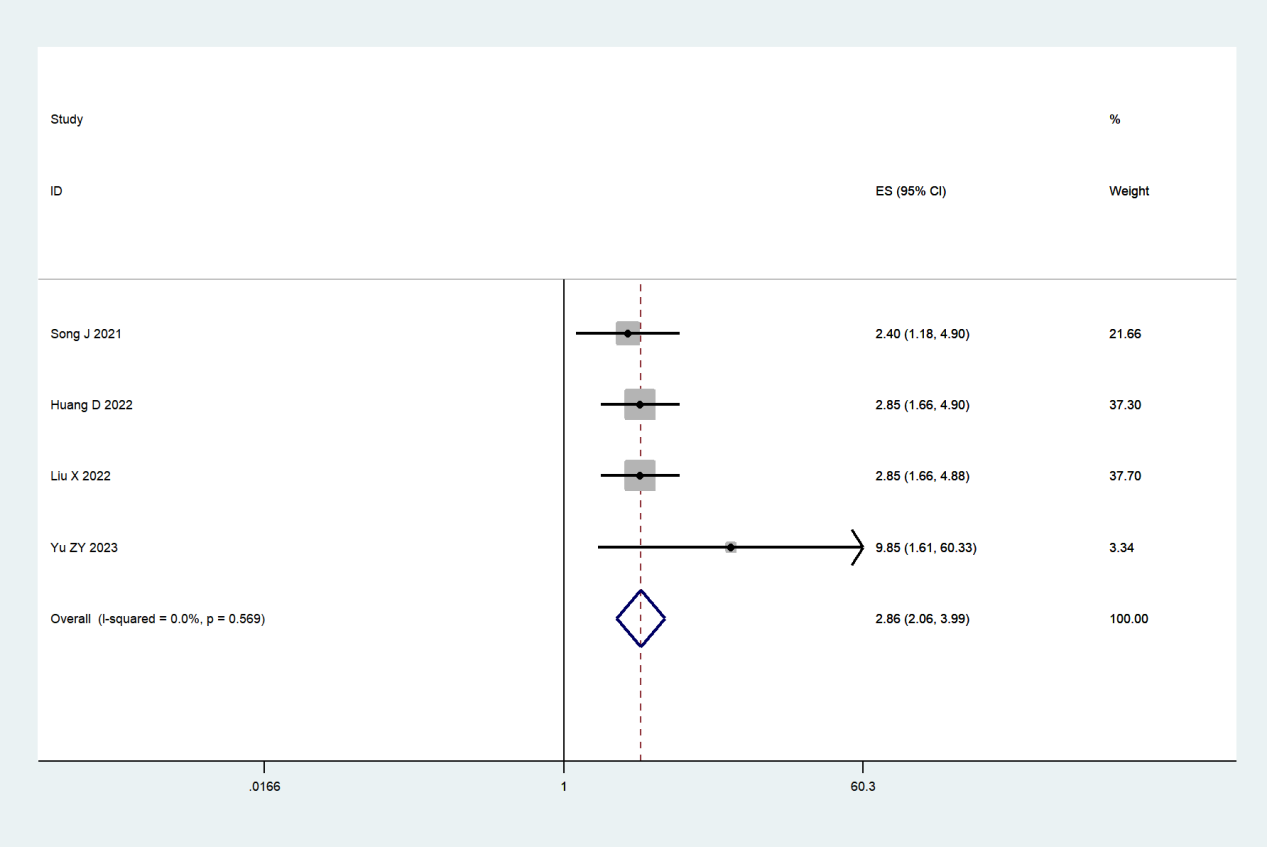


S figure 16. Forest plot of the analysis regarding severe anemia as a risk factor for NEC preterm infants. Note: NEC: necrotizing enterocolitis; ES: Odds ratio (OR).


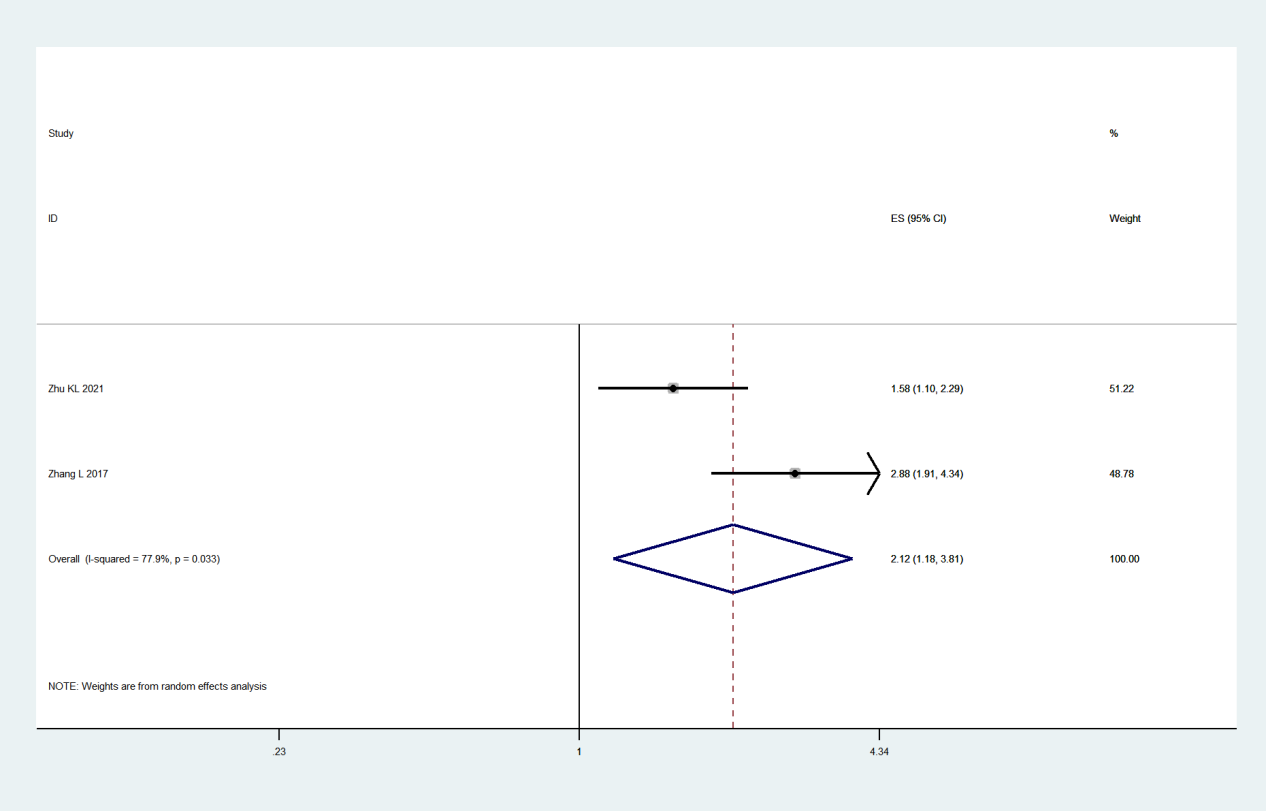


S figure 17. Forest plot of the analysis regarding history of antibiotic use as a risk factor for NEC preterm infants. Note: NEC: necrotizing enterocolitis; ES: Odds ratio (OR).


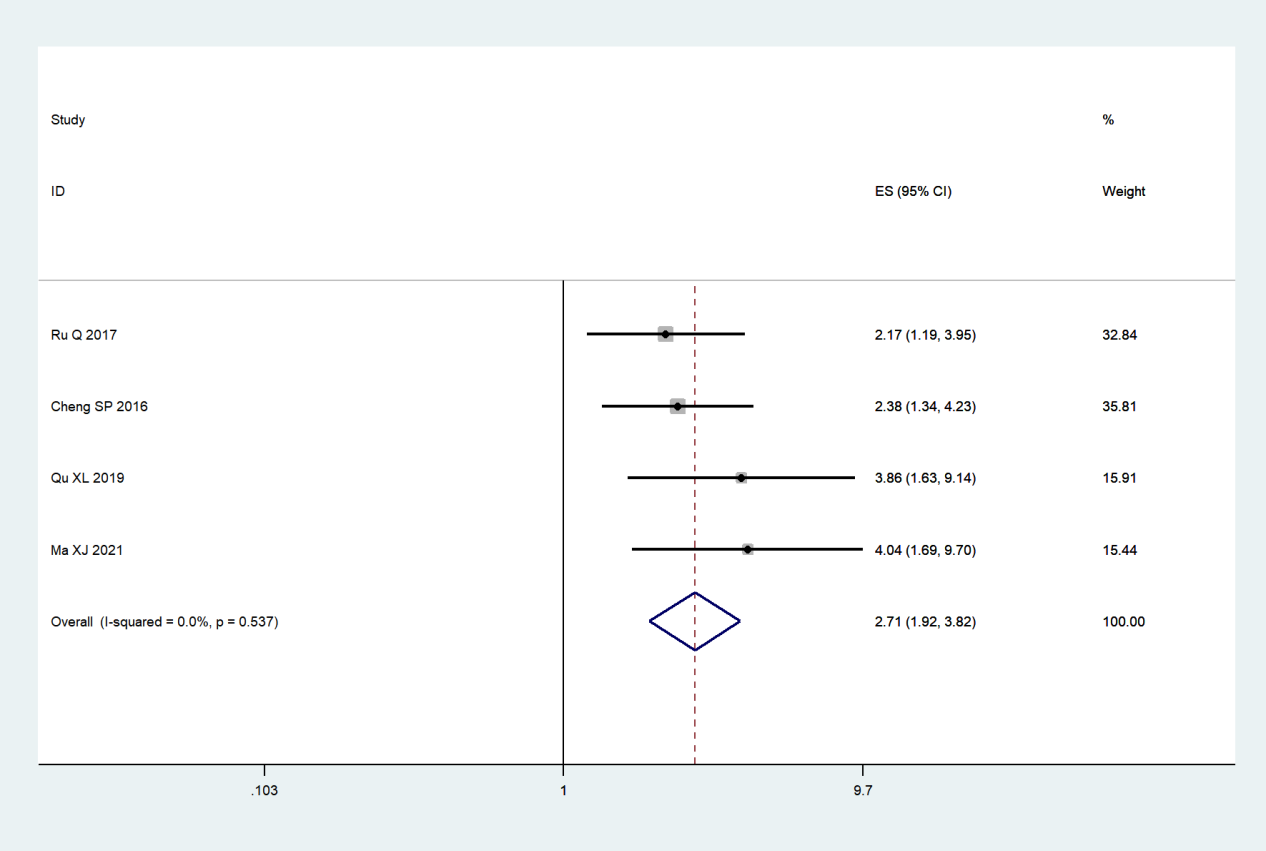


S figure 18. Forest plot of the analysis regarding intrahepatic cholestasis of pregnancy as a risk factor for NEC preterm infants. Note: NEC: necrotizing enterocolitis; ES: Odds ratio (OR).
