# Supplementary material for: Factors influencing necrotizing enterocolitis in premature infants in China: a systematic review and meta-analysis
Source: BMC Pediatr. 2024 Feb 29;24:148. doi: 10.1186/s12887-024-04607-3 (PMC10903018; doi:10.1186/s12887-024-04607-3)
Supplement: Supplementary file 6 — Additional file 6: S figure19. Funnel plot of breastfeeding. S figure20. Funnel plot of blood transfusion. S figure21. Funnel plot of oral probiotics. S figure22. Funnel plot of septicemia. [file 12887_2024_4607_MOESM6_ESM.docx]

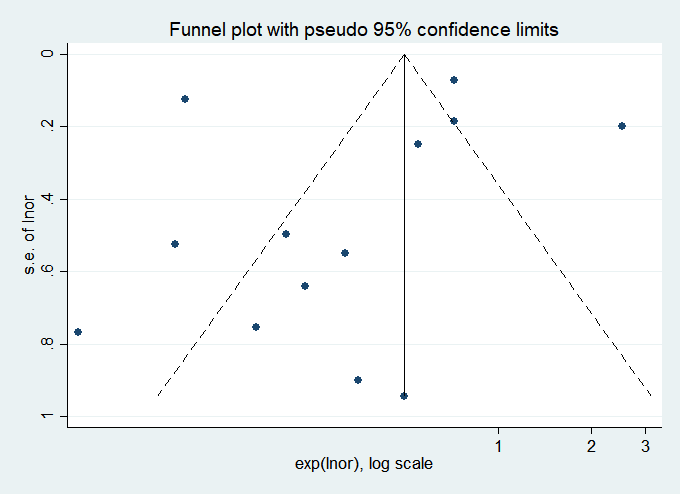


S figure19. Funnel plot of breastfeeding


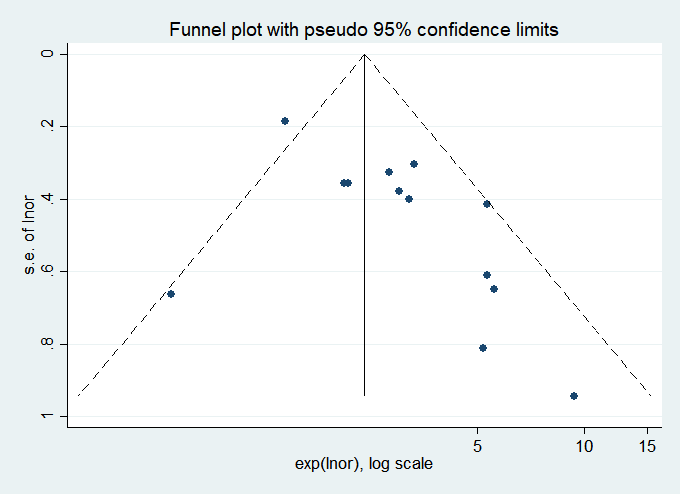


S figure20. Funnel plot of blood transfusion


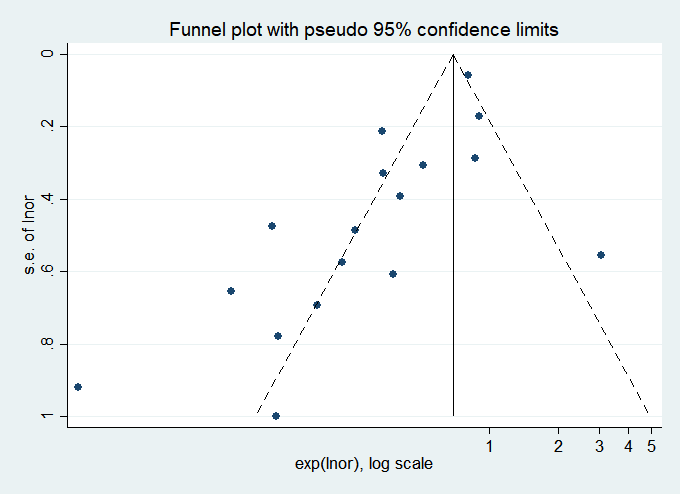


S figure21. Funnel plot of oral probiotics


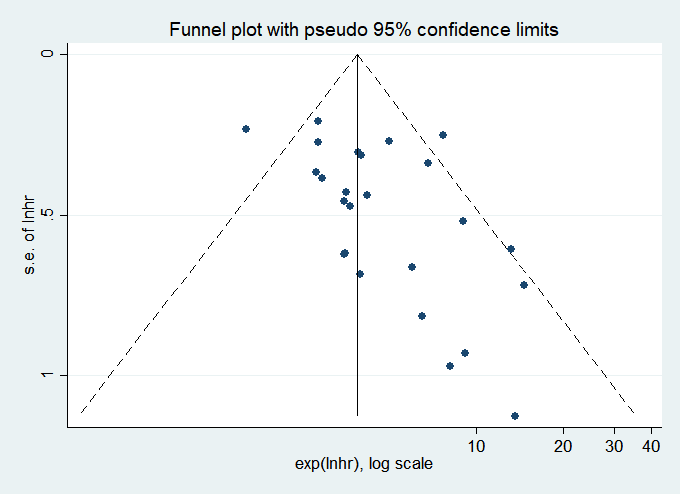


S figure22. Funnel plot of septicemia
